# Temporal dynamics of the sensorimotor convergence underlying voluntary limb movement

**DOI:** 10.1073/pnas.2208353119

**Published:** 2022-11-21

**Authors:** Tatsuya Umeda, Tadashi Isa, Yukio Nishimura

**Affiliations:** ^a^Department of Developmental Physiology, National Institute for Physiological Sciences, National Institute of Natural Sciences, Okazaki, Aichi 444-8585, Japan; ^b^Department of Integrated Neuroanatomy and Neuroimaging, Graduate School of Medicine, Kyoto University, Kyoto 606-8501, Japan; ^c^Department of Neurophysiology, National Center of Neurology and Psychiatry, Kodaira, Tokyo 187-8502, Japan; ^d^Department of Neuroscience, Graduate School of Medicine, Kyoto University, Kyoto 606-8501, Japan; ^e^Human Brain Research Center, Graduate School of Medicine, Kyoto University, Kyoto 606-8507, Japan; ^f^Institute for the Advanced Study of Human Biology (WPI-ASHBi), Kyoto University, Kyoto 606-8510, Japan; ^g^School of Life Science, The Graduate University for Advanced Studies (SOKENDAI), Hayama, Kanagawa 240-0193, Japan; ^h^Neural Prosthetics Project, Tokyo Metropolitan Institute of Medical Science, Tokyo 156-8506, Japan; ^i^Precursory Research for Embryonic Science and Technology (PRESTO), Japan Science and Technology Agency, Kawaguchi, Saitama 332-0012, Japan

**Keywords:** motor cortex, spinal reflex, voluntary movement, decoding, muscle activity

## Abstract

Descending motor drive and somatosensory feedback play important roles in modulating muscle activity. Numerous studies have characterized the organization of neuronal connectivity in which descending motor pathways and somatosensory afferents converge on spinal motor neurons as a final common pathway. However, how inputs from these two pathways are integrated into spinal motor neurons to generate muscle activity during actual motor behavior is unknown. Here, we simultaneously recorded activity in the motor cortices (MCx), somatosensory afferent neurons, and forelimb muscles in monkeys performing reaching and grasping movements. We constructed a linear model to explain the instantaneous muscle activity using the activity of MCx (descending input) and peripheral afferents (afferent input). Decomposition of the reconstructed muscle activity into each subcomponent indicated that muscle activity before movement onset could first be explained by descending input from mainly the primary motor cortex and muscle activity after movement onset by both descending and afferent inputs. Descending input had a facilitative effect on all muscles, whereas afferent input had a facilitative or suppressive effect on each muscle. Such antagonistic effects of afferent input can be explained by reciprocal effects of the spinal reflex. These results suggest that descending input contributes to the initiation of limb movement, and this initial movement subsequently affects muscle activity via the spinal reflex in conjunction with the continuous descending input. Thus, spinal motor neurons are subjected to temporally organized modulation by direct activation through the descending pathway and the lagged action of the spinal reflex during voluntary limb movement.

## Significance

Numerous investigations have identified neuronal connectivity in which descending motor pathways and somatosensory afferents converge onto spinal motor neurons. However, how volitional motor commands and reflexive somatosensory signals are integrated to generate the activity of limb muscles during actual motor behavior has not been tested. In this study, we decoded muscle activity from the activity in motor cortices and afferent neurons in behaving monkeys and revealed the temporal dynamics in which direct activation through the descending pathway from the primary motor cortex and the lagged action of the spinal reflex cooperatively modulate spinal motor neurons. The finding is an important step toward understanding a neural mechanism comprehensively explaining voluntary motor control and reflexive modulation.

Sophisticated movements of limbs in daily life involve interaction with the environment and rely on sensorimotor integration in the nervous system (1, 2). At the spinal cord level, both descending motor commands from the motor cortex and somatosensory feedback signals from peripheral afferents modulate spinal motor neurons. Extensive studies in animals and humans have provided evidence that descending motor pathways and somatosensory afferents converge on a common set of neurons in the spinal cord (3–5). The excitability of spinal interneurons or motor neurons by activation of descending projections and peripheral nerves has been directly examined in anesthetized animals. The results have elucidated the input–output structure in the spinal cord on which the descending motor and somatosensory afferent signals converge on spinal motor neurons (6). Human studies have also shown that Hoffman reflex responses are enhanced or inhibited by cortical stimulation (7, 8), revealing the integration of supraspinal influence with afferent signals from the periphery during movements. These approaches have used artificial activation of the input pathways to elucidate neuronal connectivity on spinal motor neurons. However, previous studies have not evaluated the function of actual neuronal signals through these two pathways during voluntary limb movements. Thus, it remains unknown how descending motor commands and somatosensory feedback signals are involved in the generation of muscle activity in actual motor behavior.

The contribution of descending motor drive and somatosensory feedback signals to muscle activity during actual motor behavior has been investigated by attenuating inputs during movements. For example, unexpected unloading of leg extensors in the stance phase of walking caused a reduction in muscle activity in cats and humans (9–11). Furthermore, the temporal contribution of these two signals to muscle activity has been studied by analysis of the triphasic pattern of muscle activity in rapid arm movement. Triphasic muscle activity is characterized by the first agonist activity, then antagonist activity, and then agonist activity (12, 13). Experiments to attenuate peripheral nerve transmission showed that the initial agonist muscle activity is explained by descending motor commands, whereas the subsequent antagonist and second agonist activity are explained by somatosensory feedback signals (14). However, the removal of inputs can change the balance of spinal cord circuits, leading to responses that are not normally observed. In addition, since the signals from descending pathways and peripheral afferents continuously flow into the spinal circuitry, the contribution of these signals to muscle activity should be more complicated. Therefore, the details of how these inputs cooperated to construct activity patterns of multiple limb muscles should be derived from an investigation of the descending motor drive, somatosensory feedback signals, and muscle activity during actual motor behavior.

In this study, we simultaneously recorded activity in the motor cortices (MCx), including the primary motor and premotor cortex, an ensemble of peripheral somatosensory afferents, and forelimb muscles in monkeys performing reaching and grasping movements. We constructed linear models to explain how the MCx and afferent inputs are temporally and quantitatively integrated to generate the subsequent muscle activity. Decomposition of the reconstructed muscle activity into each subcomponent indicated the temporal contribution of MCx and afferent inputs to muscle activity. Further analysis showed that descending input from the MCx drives the premovement activity of muscles to initiate movement and that the initial movement subsequently affects muscle activity by the spinal reflex in conjunction with continuous inflow of descending motor commands from the MCx.

## Results

### Descending and Afferent Inputs Account for Muscle Activity by Using a Linear Model.

**Fig. 1.**
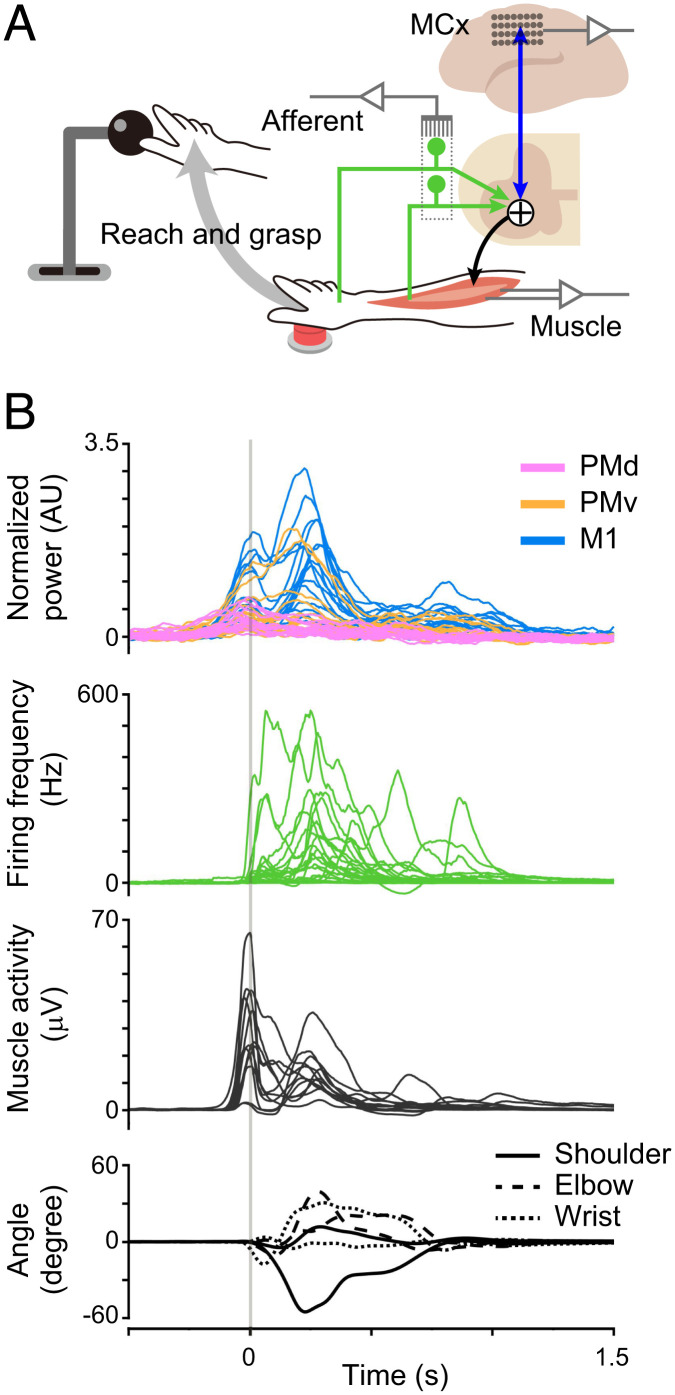
Simultaneous recording of MCx, afferent and muscle activity, and joint kinematics. (*A*) Schematic illustration of the experimental setup. (*B*) Modulation of cortical and peripheral activity in monkey T aligned to movement onset. *Top*: High-gamma cortical activity. *Second*: Instantaneous firing rate of peripheral afferents. *Third*: Forelimb muscles. *Bottom*: Joint angles. A vertical line represents the time of movement onset.

We simultaneously recorded electrocorticographic (ECoG) signals from the MCx, including primary motor (M1) and dorsal (PMd) and ventral (PMv) premotor cortices, the activity of a population of peripheral afferents at the lower cervical level (25–39 units from cervical segments C7 and C8 of monkey T, and 11–15 units from C6 and C7 of monkey C), electromyographic (EMG) signals from the forelimb muscles (12 and 10 muscles from monkeys T and C, respectively), and kinematics of the forelimb joints (wrist, elbow, and shoulder) in two monkeys, as the monkeys performed reaching and grasping movements (Fig. 1*A* and *SI Appendix*, Fig. S1) (15). An example of simultaneous multiregional recording data (monkey T, three trials) is shown in *SI Appendix*, Fig. S1. Since it is generally appreciated that cortical high-gamma activity represents the activity of the neuronal population beneath the electrode (16), we used high-gamma activity in the MCx for the analysis. Alignment of the multiregional signals to the timing of movement onset indicates the relative onset timing of these signals (Fig. 1*B*). Cortical high-gamma activity of the MCx and activity of forelimb muscles increased before movement onset, and the neuronal firing of peripheral afferents started movement-related modulation at the timing of movement onset.

**Fig. 2.**
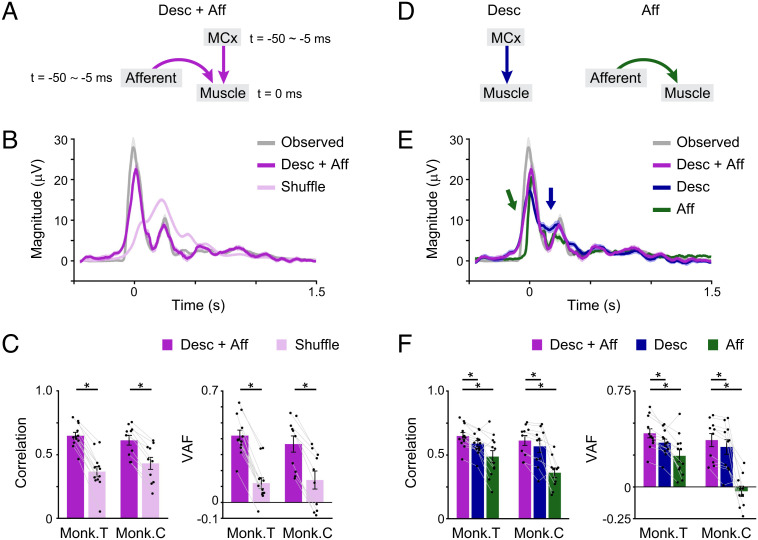
Descending and afferent inputs account for muscle activity. (*A*) Model accounting for muscle activity evoked by descending and afferent inputs. (*B*) Average modulation of the observed muscle activity, reconstruction using descending and afferent inputs, and shuffled control data aligned to movement onset. Shaded areas, SEM. (*C*) Mean reconstruction accuracy. Correlation coefficients and variance accounted fors (VAFs) between the observed and reconstructed traces (monkey T, n = 12 muscles; monkey C, n = 10 muscles; *P < 10^−5^, paired two-tailed *t* test). The superimposed bar graphs show the mean ± SEM. *P* values are described in *SI Appendix*, Table S1. (*D*) Models accounting for muscle activity evoked by descending input alone and afferent input alone. (*E*) Average modulation of the observed muscle activity, reconstruction using descending and afferent inputs, and each input aligned to movement onset. Shaded areas, SEM. Arrows indicate points that differ between the two models (dark blue, Desc + Aff vs. Desc; dark green, Desc + Aff vs. Aff). (*F*) Correlation coefficients and VAFs between the observed and reconstructed traces (monkey T, n = 12 muscles; monkey C, n = 10 muscles; *P* < 10^−4^ by one-way repeated-measures analysis of variance [ANOVA], *P < 0.01, paired two-tailed *t* test). The superimposed bar graphs show the mean ± SEM. *P* values are described in *SI Appendix*, Table S2.

We examined how descending motor drive from the MCx (descending input) and somatosensory feedback signals from peripheral afferents (afferent input) converge on spinal motor neurons to generate muscle activity. We assumed a delayed linear sum of the descending and afferent inputs as a first-order model of muscle activity (17–19). Previous studies have indicated that most MCx and peripheral afferent activities require 5–50 ms to reach spinal motor neurons (see *Materials and Methods*). Based on the conduction time, we built a linear model to explain the instantaneous muscle activity using the descending input and afferent input for 5–50 ms preceding the timing of muscle activity to be calculated (Fig. 2*A*). The model reconstructed the overall temporal pattern of muscle activity more accurately than a model built using shuffled controls (Fig. 2 *B* and *C*, paired Student’s *t* test, *P* < 10^−5^). A simple linear model accurately captures the integration of descending and afferent inputs in spinal motor neurons. The reconstruction accuracy of models built using both descending and afferent inputs was superior to that of models built using the descending input alone or afferent input alone (Fig. 2 *D*–*F*, paired Student’s *t* test, *P* < 0.01) and was superior to that of models built using descending and shuffled afferent inputs or afferent and shuffled descending inputs (*SI Appendix*, Fig. S2 *A*–*C*, paired Student’s *t* test, *P* < 0.01). In addition, the sparse linear regression algorithm pruned the inputs that were not important for the reconstruction. The proportion of descending or afferent inputs that were not pruned was higher than that of respective shuffled inputs (*SI Appendix*, Fig. S2 *D* and *E*, paired Student’s *t* test, *P* < 0.05), suggesting that both descending and afferent inputs are important for the reconstruction of muscle activity. These results indicate that descending and afferent inputs provide necessary information for the reconstruction of muscle activity.

### MCx and Afferents Sequentially Encode Muscle Activity.

**Fig. 3.**
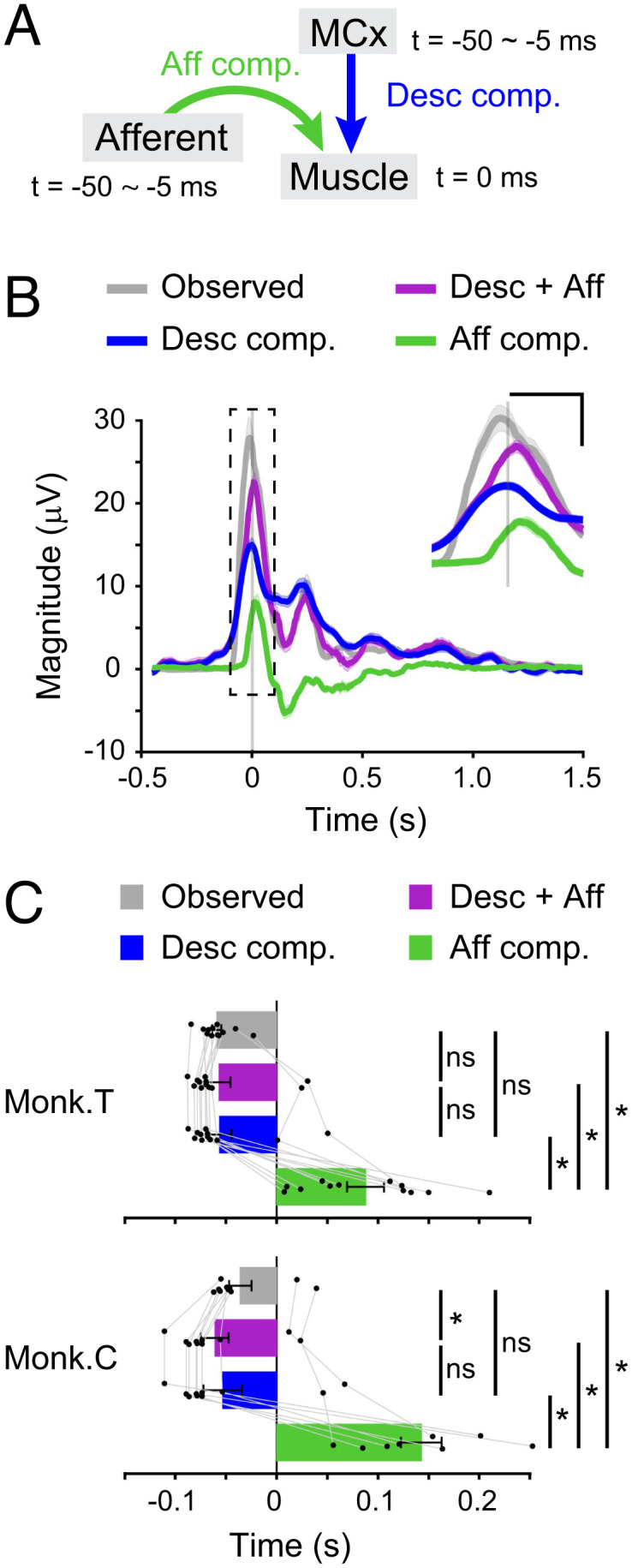
MCx and afferents sequentially encode muscle activity. (*A*) Model accounting for muscle activity evoked by descending and afferent inputs. (*B*) Average modulation of the observed muscle activity, reconstruction using descending and afferent inputs, and each subcomponent aligned to movement onset. The *Inset* shows a magnification of the graph around the movement onset indicated by the dashed line. Vertical lines represent the time of movement onset. Shaded areas, SEM. (Scale bars in the *Inset*, 0.1 s and 10 μV.) (*C*) Onset times of the observed muscle activity, reconstruction using descending and afferent inputs, and each subcomponent (monkey T, n = 12 muscles; monkey C, n = 10 muscles; *P* < 10^−4^ one-way repeated-measures ANOVA, *P < 0.05, paired two-tailed *t* test). ns, not significant. The superimposed bar graphs show the mean ± SEM. *P* values are described in *SI Appendix*, Table S3.

We assessed how each descending and afferent input contributed to the reconstructed muscle activity by calculating each subcomponent (the descending and afferent components, respectively) of the reconstructed activity (Fig. 3*A*). An example of each subcomponent in one dataset is shown in Fig. 3*B*. Descending and afferent inputs each contributed to a substantial part of muscle activity. We found a difference in temporal dynamics between the descending and afferent components; whereas the descending component (blue line) increased prior to movement onset, similar to the observed muscle activity (gray line), the afferent component (green line) increased at the timing of movement onset (Fig. 3*B*). The onset timing of the descending component (blue line) was equivalent to that of the observed muscle activity (gray line) and the reconstructed muscle activity (violet line) from both descending and afferent inputs (Fig. 3*C*, paired Student’s *t* test, *P* > 0.05). However, the afferent component (green line) increased later than the observed muscle activity (gray line), the reconstructed muscle activity (violet line) from descending and afferent inputs, and the descending component (blue line, Fig. 3*C*, paired Student’s *t* test, *P* < 0.05). We also analyzed the temporal contribution of descending and afferent inputs to the reconstructed muscle activity by building linear models within sliding time windows (*SI Appendix*, Fig. S3*A*). Before movement onset, the reconstruction accuracy of models built using both descending and afferent inputs was not different from that of models built using the descending input alone. However, after movement onset, the addition of afferent inputs led to a higher accuracy of the reconstructed muscle activity than that achieved using descending inputs alone (*SI Appendix*, Fig. S3*B*). Thus, premovement muscle activity could be explained by descending input, while muscle activity that occurred after movement onset could be explained by the sum of the descending and afferent inputs.

**Fig. 4.**
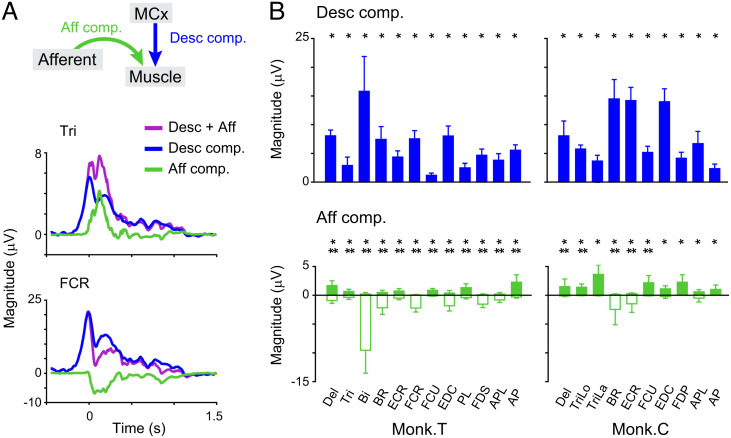
MCx and afferents differentially encode muscle activity across muscles. (*A*) Average modulation of the reconstruction using descending and afferent inputs and each subcomponent aligned to movement onset. (*B*) The size of descending and afferent components for each muscle. *Asterisks* indicate a significant difference from *0* (monkey T, n = 17 sessions; monkey C, n = 7 sessions; *P < 0.05, unpaired two-tailed *t* test for positive values; **P < 0.05, for negative values). Data are the mean ± SD. *P* values are described in *SI Appendix*, Table S4.

We next examined how descending and afferent inputs contributed to the reconstruction of the activity of each muscle. During periods in which the movement-related modulation of muscles was observed, the descending component had positive values for all muscles (blue, Fig. 4 *A* and *B*), suggesting its facilitative effect on all muscles. In contrast, afferent input had positive or negative values, which indicated the existence of both facilitative and suppressive effects on individual muscles (green, Fig. 4 *A* and *B*). Thus, descending and afferent inputs are related to subsequent muscle activity in different ways.

### M1 Primarily Encodes Muscle Activity.

**Fig. 5.**
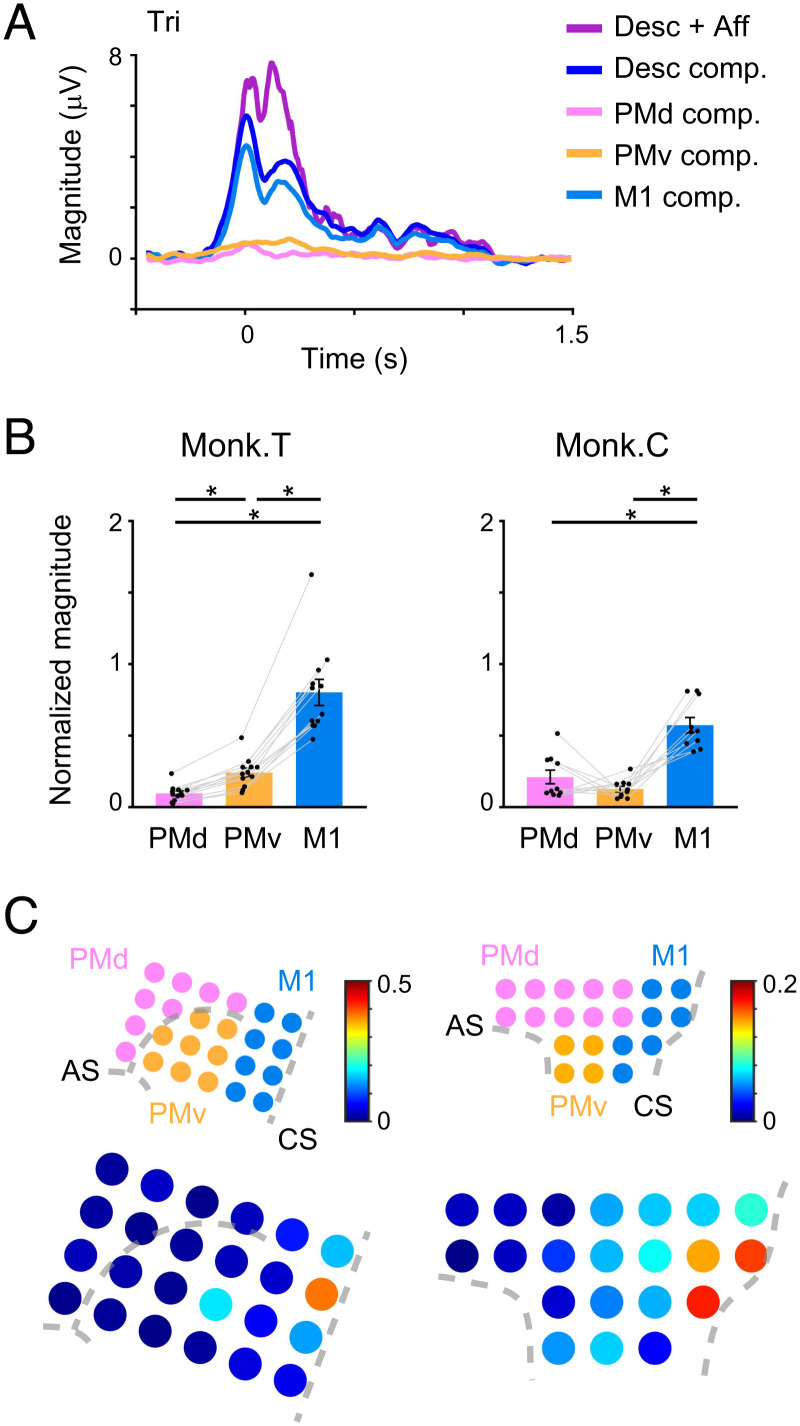
M1 is a major predictor of muscle activity. (*A*) Average modulation of the reconstruction using descending and afferent inputs and subcomponents calculated from the activity in PMd (PMd component), PMv (PMv component), and M1 (M1 component) aligned to movement onset. (*B*) The size of subcomponents calculated from the activity in each cortical area for the prediction of muscle activity (monkey T, n = 12 muscles; monkey C, n = 10 muscles; *P* < 0.05, one-way repeated-measures ANOVA, *P < 0.05, paired two-tailed *t* test). The size of each subcomponent is normalized by the size of the reconstruction using descending and afferent inputs. The superimposed bar graphs show the mean ± SEM. *P* values are described in *SI Appendix*, Table S5. (*C*) Color maps in the lower row represent the size of subcomponents calculated from the activity at each electrode for the prediction of muscle activity. Electrode positions in each motor cortical area are depicted in the upper row. The size of each subcomponent is normalized by the size of the reconstruction using descending and afferent inputs. CS, central sulcus; AS, arcuate sulcus.

We investigated to what extent the activity in each cortical area of the MCx contributed to the reconstruction of muscle activity. We calculated the descending components based on the activity in each cortical area. The descending component calculated from M1 activity was more prominent than the subcomponents calculated from PMd or PMv activity (Fig. 5 *A* and *B*, paired Student’s *t* test, *P* < 0.05). The proportion of unpruned M1 input determined by the sparse linear regression algorithm was higher than that of unpruned PMd or PMv inputs (*SI Appendix*, Fig. S4, paired Student’s *t* test, *P* < 0.05), suggesting that M1 inputs contain more of the signals needed to reconstruct muscle activity than PMd or PMv inputs. When we explored the size of subcomponents calculated using the activity measured at each electrode location, M1 sites with the largest subcomponent were found to be located just anterior to the central sulcus (Fig. 5*C*). Thus, the descending input from a subset of M1 regions rather than the PMd or PMv could primarily account for muscle activity. These results suggest that descending command from M1 is the main source for generating muscle activity.

### Afferent Effects on Muscle Activity Are Explained by the Action of the Spinal Reflex.

**Fig. 6.**
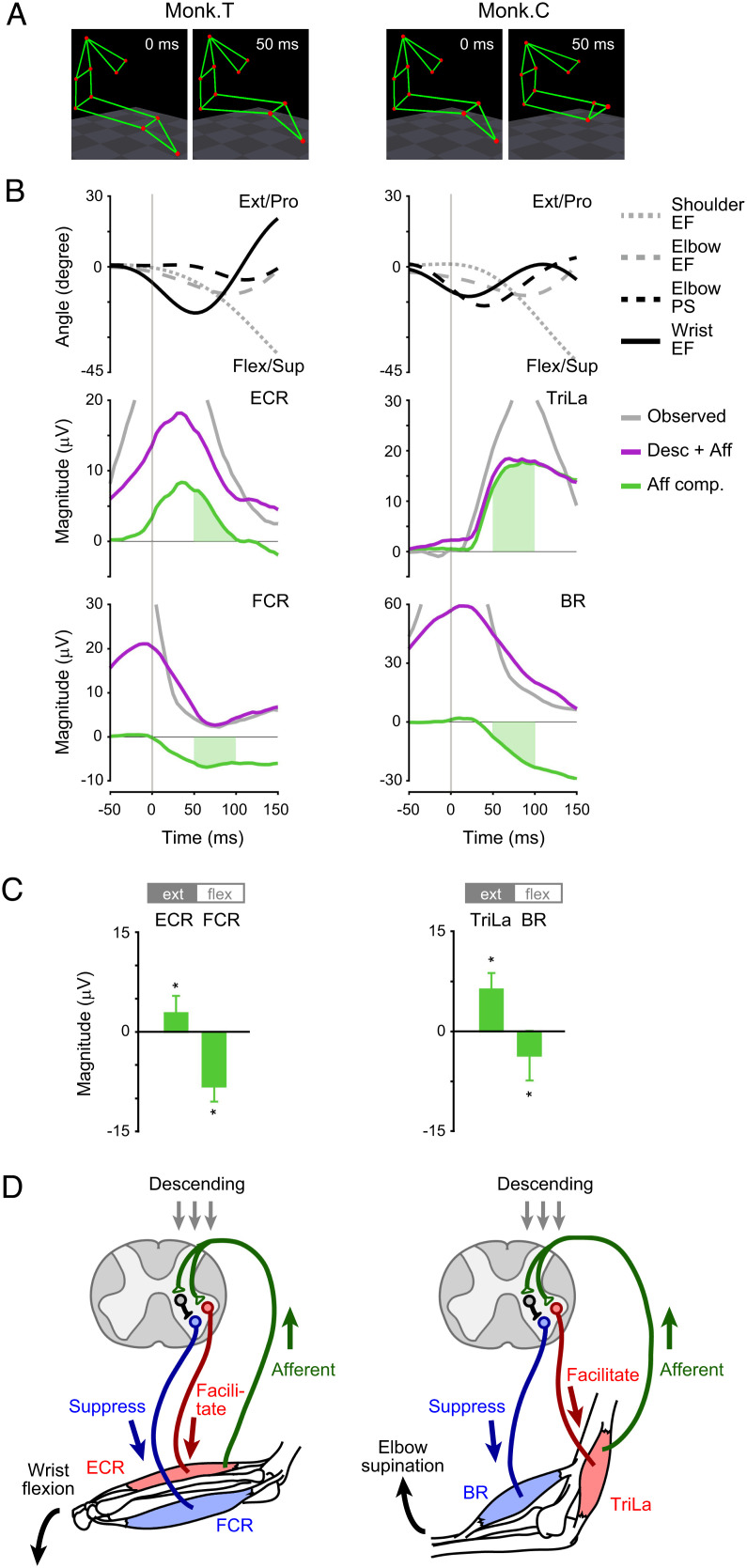
Effects of afferent input on muscle activity are accounted for by stretch reflex and reciprocal inhibition. (*A*) Stick figures showing the orientation of the forelimb and chest when the monkeys began to reach (t = 0 ms). When monkeys began to reach, monkey T flexed the wrist, and monkey C supinated and flexed the elbow. (*B*) *Top*: Forelimb joint angles. Second and Third: Average modulation of the observed muscle activity, reconstruction using descending and afferent inputs, and afferent component in the reconstruction. The vertical lines indicate the time of movement onset. (*C*) The size of afferent components for antagonistic muscle pairs (ext; extensor, flex; flexor) in a period from the beginning of the reaching movement (55–100 ms around movement onset; shown in the green area in Fig. 5*B*). *Asterisks* indicate a significant difference from *0* (monkey T, n = 17 sessions; monkey C, n = 7 sessions; *P < 0.05, unpaired two-tailed *t* test). Data are the mean ± SD. *P* values are described in *SI Appendix*, Table S6. (*D*) Schematic illustration of spinal reflex circuits and afferent effects on agonist and antagonist muscles. Agonist muscles (monkey T, ECR; monkey C, TriLa) are depicted in red, and antagonist muscles (monkey T, FCR; monkey C, BR) are depicted in blue. Afferent inputs (green arrow) induced by the stretch of agonist muscles resulted in the facilitation of agonist muscles and suppression of antagonist muscles via spinal inhibitory interneurons.

The various impacts of afferent input on different muscles are interesting. Previous studies have revealed spinal neural circuits that allow inputs from somatosensory afferents to exert different effects on motor neurons (3–5). Muscle spindles, which sense how much a muscle is lengthened, send excitatory inputs to motor neurons of the same muscle in a process, known as the stretch reflex. They also send inhibitory inputs to motor neurons of the antagonist muscles through a process, known as reciprocal inhibition (20). A previous study using a sudden stretching of the limb showed that the stretch reflex system is engaged in reaching movements (21). Therefore, we examined the relationship between the afferent component and changes in joint angle that accompany changes in muscle length. To avoid analyzing the complicated relationship between changes in joint angle and changes in muscle length during multi-joint movement, we focused on the initial movement (from 55 to 100 ms after movement onset) during which the monkeys changed the position of one or two joints. Monkey T initiated reaching by flexion of the wrist joint, and monkey C initiated reaching by supination of the elbow joint (Fig. 6*A*). We then determined the effect of the initial movement-related modulation of muscle activity via peripheral afferents as the afferent component after the initial movement.

When monkey T initiated reaching, the monkey contracted the flexor of the forearm to flex the wrist joint and stretched an extensor of the forearm, such as the extensor carpi radialis (ECR). Immediately after this initial movement, the afferent component exerted a facilitative effect on the ECR (Fig. 6 *B–D* and *SI Appendix*, Fig. S5). When monkey C initiated reaching, the monkey supinated the forearm and stretched the muscles on the lateral side, such as the elbow extensor and triceps brachii lateralis (TriLa). After this movement, the afferent input exerted a facilitative effect on the TriLa (Fig. 6 *B–D* and *SI Appendix*, Fig. S5). These relationships were consistent with the stretch reflex.

Furthermore, the afferent effect on the antagonistic muscle pairs was the opposite (Fig. 6 *B–D* and *SI Appendix*, Fig. S5). In monkey T, the afferent effect on the wrist flexor, flexor carpi radialis (FCR), which is an antagonist of the ECR, was suppressive. In monkey C, the afferent effect on an elbow flexor, the brachioradialis (BR), which is an antagonist of the TriLa, was suppressive. These findings were consistent with the conflicting inhibitory effects of afferents on spinal motor neurons, which is known as reciprocal inhibition (20). Thus, the effects of afferent inputs on muscle activity are at least partially accounted for by the action of spinal reflexes (Fig. 6*D*). These results suggest that the initial movement subsequently affects muscle activity through spinal reflex circuits.

### Sensorimotor Convergence in the Spinal Motor Neurons Has Similar Temporal Dynamics across Different Movements.

**Fig. 7.**
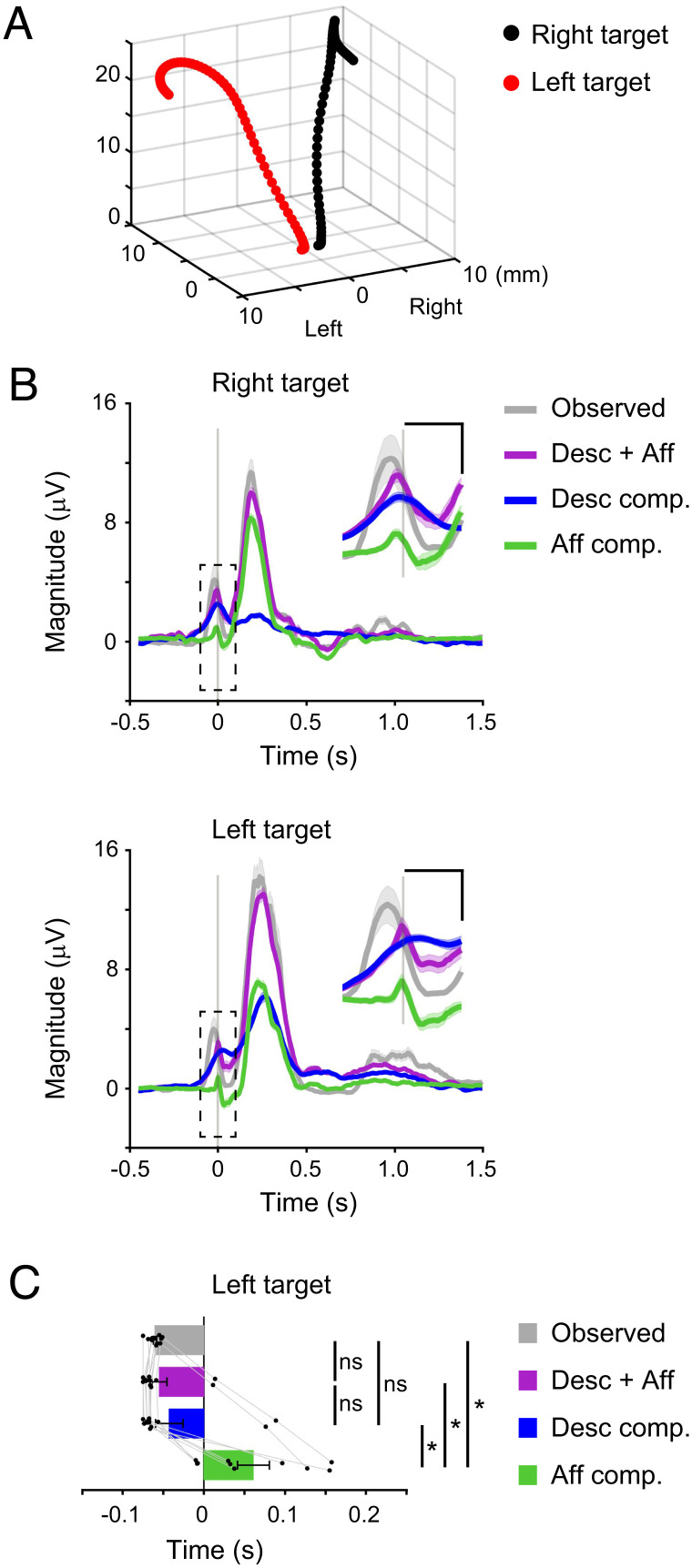
Temporal dynamics of the sensorimotor convergence in the spinal motor neurons is a common feature across different movements. (*A*) Trajectories of the right hand during the reaching and grasping movements to the right (black dots) and left targets (red dots) (−500 to 350 ms around movement onset). (*B*) Average modulation of the observed muscle activity, reconstruction using descending and afferent inputs, and each subcomponent aligned to movement onset. The *Inset* shows a magnification of the graph around the movement onset indicated by the dashed box. Vertical lines represent the time of movement onset. Shaded areas, SEM. (Scale bars in the *Inset*, 0.1 s and 2 μV.) (*C*) Onset times of the observed muscle activity, reconstruction using descending and afferent inputs, and each subcomponent (n = 12 muscles; *P* < 10^−4^ one-way repeated-measures ANOVA, *P< 0.05, paired two-tailed *t* test). ns, not significant. The superimposed bar graphs show the mean ± SEM. *P* values are described in *SI Appendix*, Table S7.

Finally, we investigated whether the temporal dynamics of the sensorimotor integration observed in the aforementioned analysis were similar for different movements. We made monkey T perform reaching and grasping movements directed toward a lever positioned more to the left than in the above experiments (Fig. 7*A*). The temporal profiles of joint angles were different during movements directed toward the two target locations (*SI Appendix*, Fig. S6*A*). Muscle activity during reaching toward the left target was generally larger than during reaching toward the right target (*SI Appendix*, Fig. S6 *B* and *C*). Specifically, the forearm flexors showed significantly higher activity levels when the monkey grasped and pulled the lever (Fig. 7*B* and *SI Appendix*, Fig. S6 *B* and *C*, paired Student’s *t* test, *P* < 10^–4^). Thus, the two movements toward different target locations differed in joint kinematics and muscle activity.

We built a linear model to explain the muscle activity using the descending and afferent inputs and calculated each subcomponent similarly. A linear model also provided an accurate prediction of muscle activity in the movements toward the left target (*SI Appendix*, Fig. S7*A*, paired Student’s *t* test, *P* < 10^–5^). Both descending and afferent inputs were important for the reconstruction of muscle activity (*SI Appendix*, Fig. S7*B*, paired Student’s *t* test, *P* < 10^–4^). The increased activity in the forearm flexors was captured by the reconstruction from the descending and afferent inputs (Fig. 7*B*, paired Student’s *t* test, *P* < 0.05) and was accounted for primarily by the increase in the descending component (Fig. 7*B* and *SI Appendix*, Fig. S6*D*, paired Student’s *t* test, *P* < 0.05). The distribution of descending and afferent components among muscles was similar to that observed during the movement to the right target (Fig. 4*B* and *SI Appendix*, Fig. S7*C*) and was consistent with the spinal reflex action (*SI Appendix*, Fig. S7*D*). The temporal profile of each component indicated that muscle activity was explained by descending input first and subsequently by the sum of the descending and afferent inputs (Fig. 7 *B* and *C*, paired Student’s *t* test, *P* < 0.05). The results suggested that the integration of descending and afferent inputs in the spinal motor neurons had qualitatively equivalent temporal dynamics across forelimb movements with different temporal profiles.

## Discussion

**Fig. 8.**
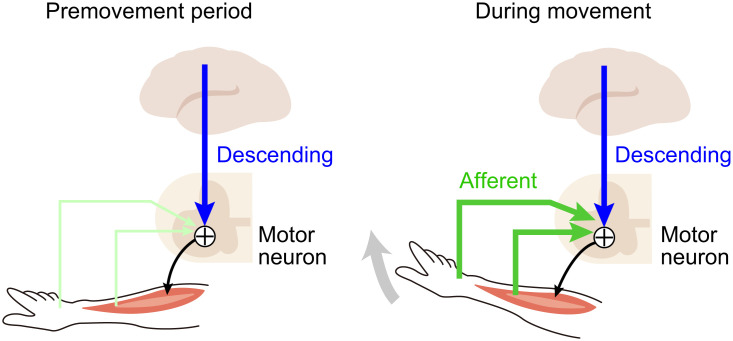
Proposed spatiotemporal dynamics of spinal motor neurons integrating descending and afferent inputs during voluntary movement. (*Left Panel*) Descending motor commands from the MCx contribute to the initiation of limb movement. (*Right Panel*) During movement, spinal motor neurons continuously receive descending motor commands from the MCx and concurrently receive sensory feedback signals from peripheral afferents via the spinal reflex.

In this study, we elucidated the convergence process through which descending motor commands and spinal reflex signals contribute to generating muscle activity during actual motor behavior by simultaneously recording the activity of the MCx, peripheral afferents, and forelimb muscles during reaching and grasping movements. Our analysis based on the linear model indicates that the descending input from the MCx accounts for the premovement activity of muscles to initiate movement, and both the descending and afferent inputs account for the muscle activity during movements. A detailed analysis of the relationship between the initial limb movement and subsequent afferent components indicates that both facilitative and suppressive relations were found to fit with the pattern of spinal reflex, suggesting that the spinal reflex circuits are involved in the modulation of muscle activity. Fig. 8 illustrates a likely mechanism for spatiotemporal dynamics of spinal motor neurons integrating descending and afferent inputs during voluntary limb movement. The results suggest that descending input contributes to the activation of muscle activity for the initiation of limb movement, and this initial movement subsequently affects muscle activity via the spinal reflex. Thus, volitional motor commands through the descending pathway and lagged somatosensory signals of the spinal reflex induced by limb movements cooperatively activate spinal motor neurons to achieve the desired movement.

### Methodological Considerations.

Stable recording of an ensemble of dorsal root ganglion (DRG) neurons during dynamic limb movements was quite challenging. Although we succeeded in recording the ensemble activity of DRG neurons in two monkeys, the duration of the recording was very short. A sufficient number of DRG neurons could only be recorded for 10 and 3 d after the electrodes were implanted in monkeys T and C, respectively. Therefore, the only way to obtain enough data to perform a linear model analysis was to have the monkeys perform a simple motor task consisting of natural movement. In addition to the ensemble activity of peripheral afferents, we recorded the activity of cortical areas and multiple forelimb muscles. The temporal order of each signal on the order of milliseconds has been clarified only by large-scale simultaneous recordings performed during movements. Furthermore, the detailed time course of the integration of the activity of the MCx and afferents in the spinal motor neurons is unclear without analysis using a linear model.

In the present study, a linear model was applied to predict muscle activity from descending and afferent inputs during reaching and grasping movements. However, it is well documented that descending motor signals from supraspinal structures modulate somatosensory inputs to alter the feedback gain of spinal sensory transmission in a state-dependent manner, representing nonlinear modulation of the activity of spinal motor neurons (22). Although we did not incorporate gain adjustment of afferent inputs into our model, we could accurately reconstruct muscle activity using the linear model (Fig. 2 *A*–*C*). Thus, the linear model would have sufficiently captured most of the neuronal mechanisms underlying the signal integration process as a previous report (17). One reason for the usefulness of the linear model is that since monkeys performed highly similar movements in this study, the descending modulation of the somatosensory inputs might be constant in such a stereotyped movement. To examine the gain modulation of somatosensory inputs, we need to record these activities in more demanding tasks that require the attention to the somatosensory inputs such as manual dexterity. If recording techniques are improved in the future to perform stable recording of neuronal activity including peripheral afferents while animals perform such demanding tasks and for longer periods of time, more complex models can be built to enable an understanding of the gain adjustment of signal transmission in the spinal cord.

*ECoG* signals provide reliable information and stable measurements for a long period. The use of ECoG electrodes enabled us to record the activity across multiple motor cortices, including the M1, PMd, and PMv. Hence, we revealed the relative contribution of each motor cortical area to the muscle activity (Fig. 5 and *SI Appendix*, Fig. S4). On the other hand, we recorded oscillatory cortical signals, whereas we recorded the unit activity of peripheral afferents. We cannot exclude the possibility that different types of neural signals exhibit different contributions to muscle activity. The analysis of descending input alone certainly provided a more accurate reconstruction of muscle activity than the analysis of afferent input alone (Fig. 2*F*). However, it would not be reasonable to simply conclude that the MCx contribute more to muscle activity than afferents. One clue that enables the quantification of the functional contribution of inputs is the pathway- and time-selective inhibition of inputs by chemogenetics or optogenetics (23). Although blocking the signaling from the MCx to the spinal cord prevents the movement itself (24, 25), inhibiting all afferent inputs might allow us to assess the contribution of each input to muscle activity. Gene transfer to all afferent neurons has not been achieved to date (26), but the problem will be overcome by the creation of transgenic primates or the development of viral vectors that can be used to infect the whole brain tissue.

### Afferent Effects on Muscle Activity.

Previous studies artificially activated peripheral afferents, such as electrical stimulation of peripheral nerves or a sudden stretching or unloading of the limb, to reveal the involvement of various spinal reflex pathways during voluntary movements (3–5, 27). Their physiological evidence is convincing, but the functional contribution of each pathway during actual movements remains obscure due to the lack of data on peripheral afferent activity in behaving animals. We achieved simultaneous recording of neural activity in multiple regions, including a population of peripheral afferents (Fig. 1 and *SI Appendix*, Fig. S1), allowing us to estimate the effects of afferent inputs on muscle activity using a linear model. As a result, we found that the initial effects of afferent inputs on some muscles are represented as a combined action of the stretch reflex and reciprocal inhibition (Fig. 6 and *SI Appendix*, Fig. S5). Our results regarding facilitative effects on muscle activity are consistent with previous evidence that the Ia monosynaptic stretch reflex system is engaged in reaching movements (21). On the other hand, somatosensory feedback signals through afferents with other modalities, such as Golgi tendon organs and cutaneous receptors, are also known to act on spinal motor neurons and are involved in the modulation of muscle activity (9, 11, 28–30). Studies using a sudden unloading of the limb showed that inputs from Golgi tendon organs modulate muscle activity during locomotion in a state-dependent manner (5, 29), but its state dependency makes the analysis difficult. How signals of these other modalities are involved in the generation of muscle activity during actual motor behavior is a subject for future research.

Neurotransmission from peripheral afferents to the spinal cord during voluntary movements is strongly inhibited by presynaptic inhibitory mechanisms (31–33). Despite powerful presynaptic inhibition, more than half of the transmission is still functional during voluntary movements (32). Elimination of afferent transmission showed that afferent inputs typically produce 30–50% of the force (34, 35). A large body of experimental evidence indicates that the spinal reflex is appreciably responsible for the generation of muscle activity in walking (9–11, 27) and arm and hand movements (12, 14, 21, 36). Our study also shows that a substantial part of muscle activity could be explained by afferent inputs (Fig. 4). The size of the afferent component in our analysis is equivalent to the extent to which peripheral afferents contributed to the background muscle activity during walking, as shown in previous reports (34, 35).

### Descending Effects on Muscle Activity.

Our analysis showed that descending inputs had facilitative effects on all muscles (Fig. 4). However, experiments that examined corticospinal connectivity in monkeys have shown that corticospinal neurons in the anterior bank of the M1 have both facilitative and suppressive effects on muscle activity (6, 37, 38). These effects are mediated by the mono- and disynaptic connections to spinal motor neurons. Muscles that are coactive with corticospinal neurons receive excitatory inputs from those neurons, while antagonist muscles that are not coactive receive inhibitory inputs via inhibitory interneurons. It is usually difficult to detect suppressive effects using spike-triggered averaging because corticospinal neurons are silent when antagonist muscles are activated or are activated when antagonist muscles are silent (37, 39). Thus, the present analysis that computes linear relationships between the MCx and muscle activity may also have difficulty in detecting suppressive effects for the same reason. In addition, previous studies have shown that the magnitude of polysynaptic inhibitory transmission from the MCx to spinal motor neurons is generally smaller than that of more direct facilitatory transmission (37, 40). ECoG signals represent the sum of the activity of the neuronal population in the vicinity of the recording electrode (41). Therefore, our analysis is more likely to capture the facilitative effect as a whole. On the other hand, afferent inputs had a suppressive impact on some muscles. During the early stages of reaching movements, one or two joints moved in a particular direction that activated only a subset of peripheral afferents. Hence, some muscles may receive reciprocal inhibitory inputs strongly rather than facilitative ones.

## Materials and Methods

### Monkeys.

We used one adult male monkey (monkey T, weight 6–7 kg, *Macaca fuscata*) and one adult female monkey (monkey C, weight 5–6 kg, *Macaca mulatta*). The experiments were approved by the experimental animal committee of the National Institute of Natural Sciences. The animals were cared for and treated humanely in accordance with the *National Institutes of Health Guide for the Care and Use of Laboratory Animals*. The dataset is the same as the dataset used in our previous study (15). However, the research focus in this work is completely different because a previous study investigated the activity of the primary somatosensory cortex (*SI Appendix*, Fig. S1).

### Surgery.

All surgical procedures were performed using sterile techniques, while the animal was anesthetized with 1–2% isoflurane (*monkeys T* and *C*). Dexamethasone, atropine, and ampicillin were administered preoperatively; ampicillin and ketoprofen were given postoperatively. For EMG recording, we implanted pairs of Teflon-insulated wire electrodes (AS631; Cooner Wire) into the forelimb muscles on the right side. We used the activity in the deltoideus posterior (Del), triceps brachii (Tri), biceps brachii (Bi), brachioradialis (BR), extensor carpi radialis (ECR), flexor carpi radialis (FCR), flexor carpi ulnaris (FCU), extensor digitorum communis (EDC), palmaris longus (PL), flexor digitorum superficialis (FDS), abductor pollicis longus (APL), and adductor pollicis (AP) of monkey T and the Del, triceps brachii longus (TriLo), triceps brachii lateralis (TriLa), BR, ECR, FCU, EDC, flexor digitorum profundus (FDP), APL, and AP of monkey C. To record ECoG signals from the MCx, we implanted a 32 - channel grid electrode array (Unique Medical) with a diameter of 1 mm and an interelectrode distance of 3 mm beneath the dura mater over the sensorimotor cortex. We placed the ground and reference electrodes over the ECoG electrode so that they contacted the dura. We implanted two multi-electrode arrays (Blackrock Neurotech) into the DRG at the cervical level (monkey T: C7 and C8; monkey C: C6 and C7) on the right side to record afferent signals.

### Behavioral Task.

All monkeys were operantly conditioned to perform a reach-to-grasp task with the right hand (Fig. 1*A*). After putting its hand on a home button for 2–2.5 s, the monkey reached for a lever and pulled it to receive a reward. For the analysis of monkey T, the lever was placed in two target locations. Data were acquired from 17 and four sessions of reaching toward the right and left targets placed in front of the body, respectively, and the left target was placed 18 cm away from the right target. For the analysis of monkey C, in seven sessions, the lever was placed at the same position as that used for the right target during the analysis of monkey T.

### Recordings.

All neural and muscular signals were recorded simultaneously using a data acquisition system (Plexon). EMG signals were amplified using amplifiers (AB-611J; Nihon Kohden); they were sampled at 2,000 Hz in monkey T and at 1,000 Hz in monkey C at a gain of ×1,000–2,000. We applied a second-order Butterworth bandpass filter (1.5–60 Hz) to the signals, rectified the filtered signals, resampled the signals at 200 Hz, and smoothed the resampled signals using a moving window of 11 bins.

The ECoG signals were amplified using a multichannel amplifier (Plexon MAP system; Plexon) at a gain of ×1,000 and sampled at 2,000 Hz in monkey T and at 1,000 Hz in monkey C. We applied a second-order Butterworth bandpass filter (1.5–240 Hz) to the signals, computed a short-time *fast Fourier transform* on moving 100-ms windows of the filtered signals at a 5-ms step, normalized the power to the average power in each session, and calculated the average power in the high-gamma bands (high-gamma 1, 60–120 Hz; high-gamma 2, 120–180 Hz). We considered the high-gamma power of the ECoG signals to be representative of neural activity in cortical areas (16).

The peripheral afferent activities were initially amplified at a gain of ×20,000 and sampled at 40 kHz (Plexon MAP system; Plexon). We extracted filtered waves (150–8,000 Hz) above an amplitude threshold, sorted the thresholded waves using semiautomatic sorting methods (Offline Sorter; Plexon), and performed manual verification. We isolated 25–39 units in monkey T and 11–15 units in monkey C. We convolved the inversion of the interspike interval using an exponential decay function whose time constant was 50 ms and resampled the firing rate at 200 Hz.

We calculated the movement-related modulation of the EMG signals, the ECoG signals, and the peripheral afferent activity before analyzing the data. We first calculated the baseline activity by averaging the activity from −1250 to −750 ms around movement onset. We then subtracted the baseline activity from the preprocessed activity. We used movement-related modulation throughout the premovement and movement periods (−500 to 1,500 ms around movement onset) as a single trial for further analysis.

We recorded the times at which the animals released the home button, pulled the lever, and pushed the home button.

We recorded forelimb movements using an optical motion capture system with 12 cameras (Eagle-4; Motion Analysis). The spatial positions of ten reflective markers attached to the surface of the forelimbs and body were sampled at 200 Hz. We calculated the joint angles flexion/extension (FE) of the shoulder, adduction/abduction (AA) of the shoulder, FE of the elbow, pronation/supination (PS), FE of the wrist, and radial/ulnar (RU) of the wrist (15).

### Sparse Linear Regression.

Neural ensemble activity in the MCx accounts well for muscle activity when a linear model is used (19, 42, 43). We examined whether integration of the descending signals from the MCx and somatosensory signals from peripheral afferents in spinal motor neurons can also be represented as a linear relationship. We modeled muscle activity as a weighted linear combination of high-gamma activity in the MCx and/or neuronal activity of peripheral afferents using multidimensional linear regression, as follows:


[1] $$y_{j,T}\left(t\right)=\;\sum_{k,l}w_{j,k,l}\times x_{k,T}(t+l\delta ),$$

where *y*_j,*T*_(*t*) is a vector of the EMG activity of muscle *j* (12 and 10 muscles for monkeys T and C, respectively) at time index *t* in trial *T*, *x*_*k,T*_(*t* + *lδ*) is an input vector of the peripheral afferent or cortical signal *k* at time index *t* and lag time *lδ* (*δ* = 5 ms, *l* = −10 to −1) in trial *T*, and *w_j,k,l_* is a vector of weights on the peripheral afferent or cortical signal *k* at lag time *lδ*. We applied a Bayesian sparse linear regression algorithm that introduces sparse conditions for the unit/channel dimension (44). As we examined how the combined activity in the MCx and/or peripheral afferents influenced subsequent muscle activity, lag time *lδ* (Eq. **1**) was set to negative values. To represent the direct effect of the MCx on muscle activity through the descending pathway (descending input), we used activity in the MCx from −50 to −5 ms to reconstruct muscle activity at time 0 for the following reasons. Averaging the muscle activity triggered at the spiking activity of M1 neurons shows postspike facilitation with a latency of 6.7 ms (39). Accordingly, we set 5 ms as the shortest lag time. The weighted sum of M1 neuronal activity accurately accounted for muscle activity at a lag time of 40–60 ms (45). However, it is possible that the MCx has some effect on muscle activity through the spinal reflex arc within approximately 50 ms. To avoid the influence of the spinal reflex arc, we set 50 ms as the longest lag time. To represent the effect of peripheral afferents on muscle activity (afferent input), we used the activity in the peripheral afferents from −50 to −5 ms to reconstruct muscle activity at time 0 for the following reasons. Averaging the muscle activity triggered at the spiking activity of peripheral afferents showed postspike facilitation with a latency of 5.8 ms (46). Thus, we set 5 ms as the shortest lag time. The transcortical long-latency reflex is detected as a burst of muscle activity occurring 50–100 ms following an imposed limb displacement (47). To avoid the influence of the transcortical long-latency reflex on muscle activity, we set 50 ms as the longest lag time. To examine the contribution of the descending and afferent inputs to muscle activity within short time windows, we built a model that reconstructed muscle activity from a weighted linear combination of descending and afferent inputs within an overlapping, sliding time window of 500 ms.

To compute the contribution of each descending and afferent input to the reconstruction of muscle activity, we calculated each component of the reconstructed activity using the relevant input and the respective weight values in a decoding model built using the combined inputs. For example, the descending component was calculated as follows:


[2] $$\begin{array}{c} y\_{\mathrm{Desc}}_{j,T}\left(t\right)=\;\sum_{k,l}w_{j,k,l}\times x\_{\mathrm{Desc}}_{k,T}\left(t+l\delta \right), \end{array}$$

where *y_Desc_j,T_*(*t*) is a vector of the descending component at muscle *j* at time index *t* in trial *T*, *x_Desc_k,T_*(*t *+* lδ*) is an input vector of the cortical signal *k* at time index *t* and lag time *lδ* in trial *T*, and *w_j,k,l_* is derived from a vector of weights in Eq. **1** but with the weights assigned to peripheral afferents removed. We also calculated subcomponents using the activity in each cortical area or each electrode in a similar manner.

### Data Analysis.

We analyzed data from 21 and seven sessions of 10 min for monkeys T and C, respectively. All sessions contained no fewer than 129 trials. We built models designed to reconstruct the temporal changes in the EMG signals using a partial dataset (training dataset) and tested them using the remainder of the same dataset (test dataset) in each session. One hundred and eight trials were selected randomly as a training dataset, and 21 trials were selected randomly from the remaining trials as the test dataset. To assess the model, we calculated the correlation coefficients between the observed data and their reconstruction in the test dataset. We also calculated the variance accounted for (VAF) as follows:


[3] $$VAF=1-\frac{\sum {y(t)-f(t)}^{2}}{\sum {\left(y(t)-\bar{y(t)}\right)}^{2}},$$

where *y*(*t*) is a vector of the actual activity in muscles at time index *t*, $\overset{-}{y(t)}$ is the mean of *y*(*t*), and *f*(*t*) is the reconstructed activity at time index *t*. We performed sixfold cross-validation in the analysis of each session and used averaged values for the analysis. We then calculated the averaged values for each muscle from data obtained from 17 (monkey T toward the right target), four (monkey T toward the left target), and seven (monkey C) sessions. In control analyses of the reconstruction performed by the model, we created surrogate training datasets in which we shuffled the temporal profiles of the inputs independently across different blocks to generate a model and subsequently tested the model using the unshuffled data. To assess the accuracy of the reconstruction using sliding time windows (*SI Appendix*, Fig. S3), we calculated the VAF using traces averaged over 21 trials. We performed sixfold cross-validation in the analysis of each session and used median values for the analysis. We then calculated the average values in each muscle from data obtained from 17 (monkey T toward the right target) or seven (monkey C) sessions.

To obtain the onset time of the observed muscle activity or the reconstruction, we first calculated the average of the aligned waveforms in the test dataset (Figs. 3*C* and 7*C*). We then defined one-fifth of the maximum amplitude of the observed muscle activity from 250 ms before to 250 ms after movement onset as a threshold. If the activity or the reconstruction exceeded the threshold in five consecutive bins, the first of these bins was set as the onset. We calculated the average onset values observed in six test datasets in one session and obtained their average values over all sessions. We also obtained a period in which movement-related modulation of muscles was observed. The onset time of muscle activity was obtained as described above using all datasets (−59.2 ± 15.7 ms (mean ± SD) for monkey T during reaching toward the right target, −60.4 ± 7.1 ms for monkey T during reaching toward the left target, and −35.9 ± 35.3 ms for monkey C). To obtain the offset time, we calculated the average of the aligned waveforms of the muscle activity and analyzed data obtained 500 ms after the onset of movement. If the averaged data values were below the same threshold that was used to calculate the onset time in five consecutive bins, the first of these bins was set as the offset (1,012 ± 28 ms for monkey T toward the right target, 1,034 ± 88 ms for monkey T toward the left target, and 1,137 ± 118 ms for monkey C). The calculated onset and offset times corresponded well with those determined by visual inspection. We calculated the temporal mean of positive or negative (monkeys T and C) values of each component during the period in which movement-related modulation of muscles was detected (monkey T, −100 to 1,150 ms around movement onset; monkey C, −100 to 1,300 ms) (Fig. 4 and *SI Appendix*, Fig. S7*C*). We assessed whether the positive or negative values deviated from zero using Student’s *t* test. The time at which the wrist joint angle initially peaked along the flexion/extension direction in monkey T was 50.9 ± 10.6 ms (mean ± SD) for the right target and 53.8 ± 2.5 ms for the left target, and the times at which the wrist joint angle initially peaked along the flexion/extension direction and at which the elbow joint angle initially peaked along the pronation/supination direction in monkey C were 31.4 ± 5.6 ms and 47.1 ± 2.7 ms, respectively (Fig. 6*C*). We calculated the temporal mean of each component during the period from the beginning of the reaching movement (from 55 to 100 ms around movement onset) (Fig. 6*D* and *SI Appendix*, Figs. S5 and S7*D*). We statistically verified whether the temporal mean deviated from zero.

### Statistical Analysis.

We used the paired or unpaired Student’s *t* test. When comparing more than two group means, we first assessed the data using *one-way ANOVA*. The alpha level of significance was set at 0.05. The data are expressed as the mean ± SEM or the mean ± SD. We used MATLAB R2020a (MathWorks) for statistical analysis. No statistical methods were used to predetermine the sample size. However, the sample sizes followed published standards.

## Supplementary Material

Appendix 01 (PDF)

## Data Availability

The codes and datasets are available in the Zenodo repository (https://zenodo.org/deposit/7100949) (48).
