# Supplementary material for: Temporal dynamics of the sensorimotor convergence underlying voluntary limb movement
Source: Proc Natl Acad Sci U S A. 2022 Nov 21;119(48):e2208353119. doi: 10.1073/pnas.2208353119 (PMC9860324; doi:10.1073/pnas.2208353119)
Supplement: Supplementary file 1 — Appendix 01 (PDF) [file pnas.2208353119.sapp.pdf]

PNAS

Supporting Information for
Temporal dynamics of the sensorimotor convergence underlying voluntary limb movement

Tatsuya Umeda, Tadashi Isa, and Yukio Nishimura

Tatsuya Umeda, Yukio Nishimura
Email: umeda.tatsuya.4n@kyoto-u.ac.jp (T.U.), nishimura-yk@igakuken.or.jp (Y.N.)

This PDF file includes:

Figures S1 to S7
Tables S1 to S19

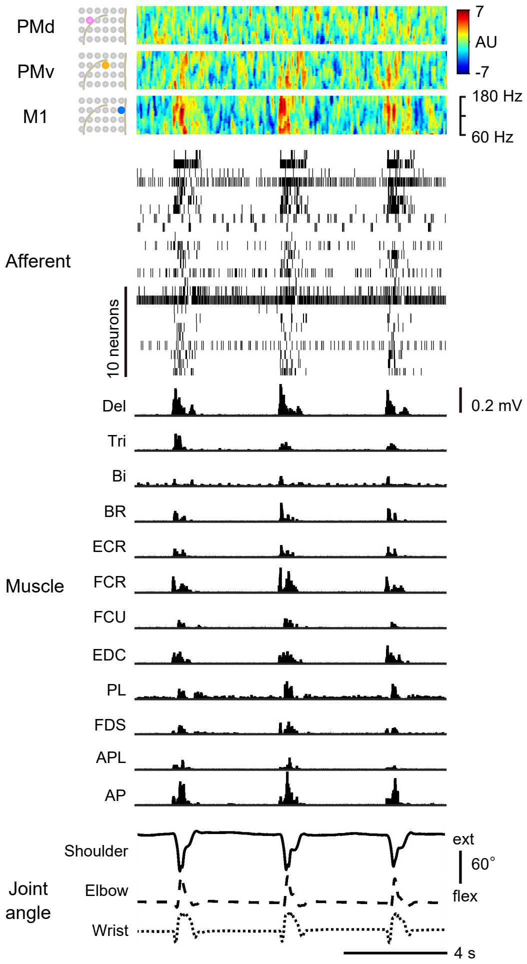

Fig. S1. Simultaneous recording of cortical and peripheral activity. Representative simultaneous recordings obtained in three trials with monkey T. *First panel*: Power spectrograms recorded in the MCx. *Second panel*: Raster plots of peripheral afferent activity. *Third panel*: Activity of forelimb muscles. *Fourth panel*: Forelimb joint angles along the extension-flexion axis.

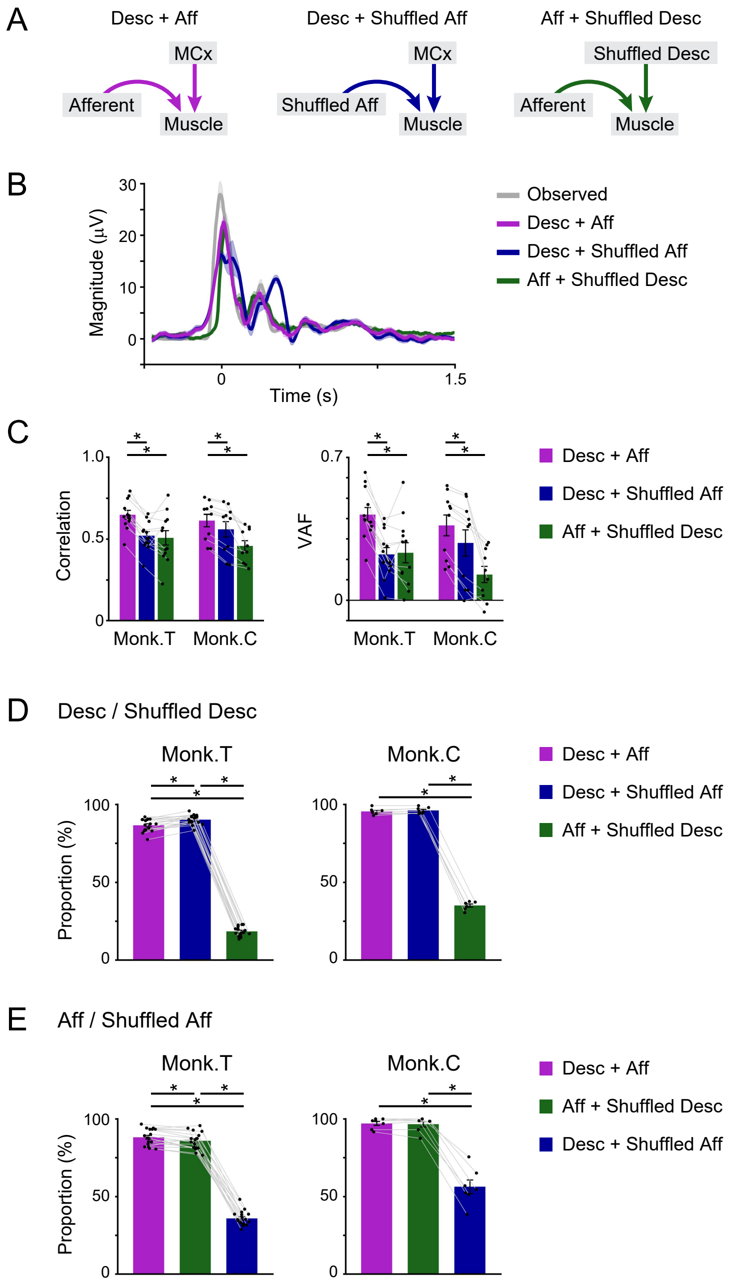

Fig. S2. Descending and afferent inputs are necessary for the reconstruction of muscle activity. (*A*) Models accounting for muscle activity evoked by descending and afferent inputs, descending and shuffled afferent inputs, or afferent and shuffled descending inputs. (*B*) Average modulation of the observed muscle activity, reconstruction using descending and afferent inputs, descending and shuffled afferent inputs, and afferent and shuffled descending inputs aligned to movement onset. Shaded areas, SEM. (*C*) Correlation coefficients and VAFs between the observed and reconstructed traces (monkey T, n = 12 muscles; monkey C, n = 10 muscles; $P < 10^{-4}$, one-way repeated-measures analysis of variance [ANOVA], *$P < 0.001$, paired two-tailed *t*-test). The superimposed bar graphs show the mean ± SEM. *P* values are described in Table S8. (*D*) The proportion of descending or shuffled descending inputs selected by the sparse linear regression algorithm in the reconstruction of muscle activity (purple, models using descending and afferent inputs; dark blue, models using descending and shuffled afferent inputs; dark green, models using afferent and shuffled descending inputs; monkey T, n = 17 sessions; monkey C, n = 7

sessions; *P* < 0.05, one-way repeated-measures ANOVA, **P* < 0.05, paired two-tailed *t*-test). The superimposed bar graphs show the mean ± SEM. *P* values are described in Table S9. (*E*) The proportion of afferent or shuffled afferent inputs selected from the total number of inputs by the sparse linear regression algorithm in the reconstruction of muscle activity (purple, models using descending and afferent inputs; dark green, models using afferent and shuffled descending inputs; dark blue, models using descending and shuffled afferent inputs; monkey T, n = 17 sessions; monkey C, n = 7 sessions; *P* < 0.05, one-way repeated-measures ANOVA, **P* < 0.05, paired two-tailed *t*-test). The superimposed bar graphs show the mean ± SEM. *P* values are described in Table S10.

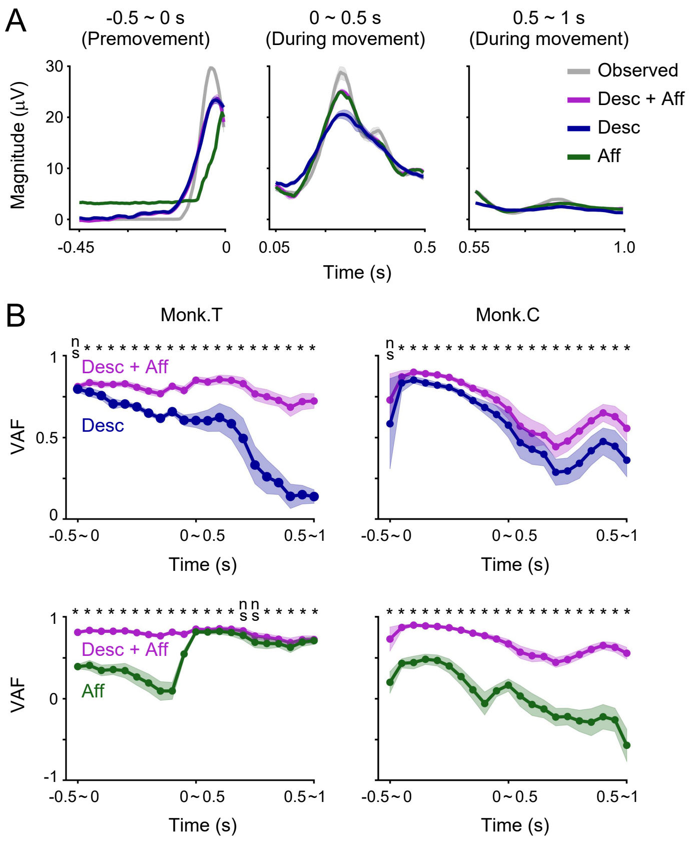

Fig. S3. Descending, not afferent, input contributes to the reconstruction of muscle activity before movement onset. (*A*) Average modulation of the observed muscle activity, reconstruction using descending and afferent inputs, and each input in three 0.5-s time windows (premovement period, -0.5 to 0 s around movement onset; during movement, 0 to 0.5 s around movement onset; and 0.5 to 1.0 s around movement onset). Shaded areas, SEM. (*B*) Mean reconstruction accuracy of the reconstructed traces for each 0.5-s sliding window ((*upper panel*), Desc + Aff vs. Desc; (*lower panel*), Desc + Aff vs. Aff) (monkey T, n = 12 muscles; monkey C, n = 10 muscles; **P* < 0.05, paired two-tailed *t*-test with Holm–Bonferroni correction). ns, not significant. Shaded areas, SEM. *P* values are described in Table S11.

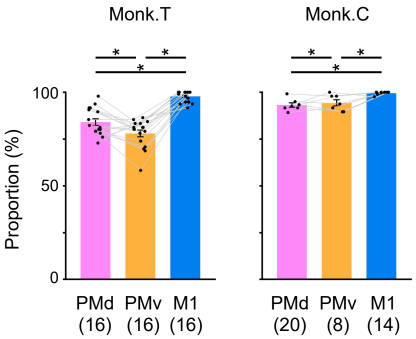

Fig. S4. M1 inputs contain more signals needed for the reconstruction of muscle activity. The proportion of PMd, PMv, and M1 inputs selected from the total inputs for the respective cortical area by the sparse linear regression algorithm in the reconstruction of muscle activity (monkey T, n = 17 sessions; monkey C, n = 7 sessions; $P < 0.005$, one-way repeated-measures ANOVA, *$P < 0.05$, paired two-tailed t-test). The total number of inputs for each cortical area is denoted under the name of each cortical area. The superimposed bar graphs show the mean ± SEM. P values are described in Table S12.

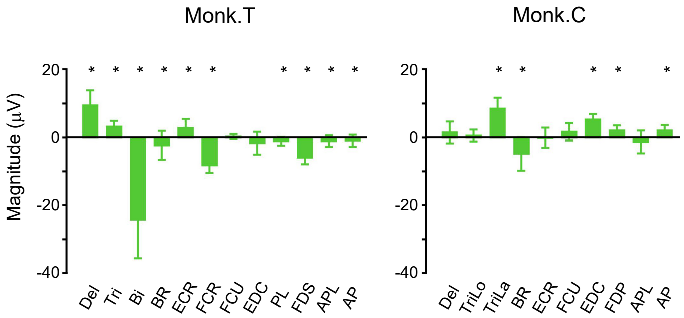

Fig. S5. The size of afferent components for each muscle in a period from the beginning of the reaching movement (55 to 100 ms around movement onset; shown in the green area in Fig. 5B). Asterisks indicate a significant difference from 0 (monkey T, n = 17 sessions; monkey C, n = 7 sessions; *$P < 0.05$, unpaired two-tailed *t*-test). Data are the mean ± SD. *P* values are described in Table S13.

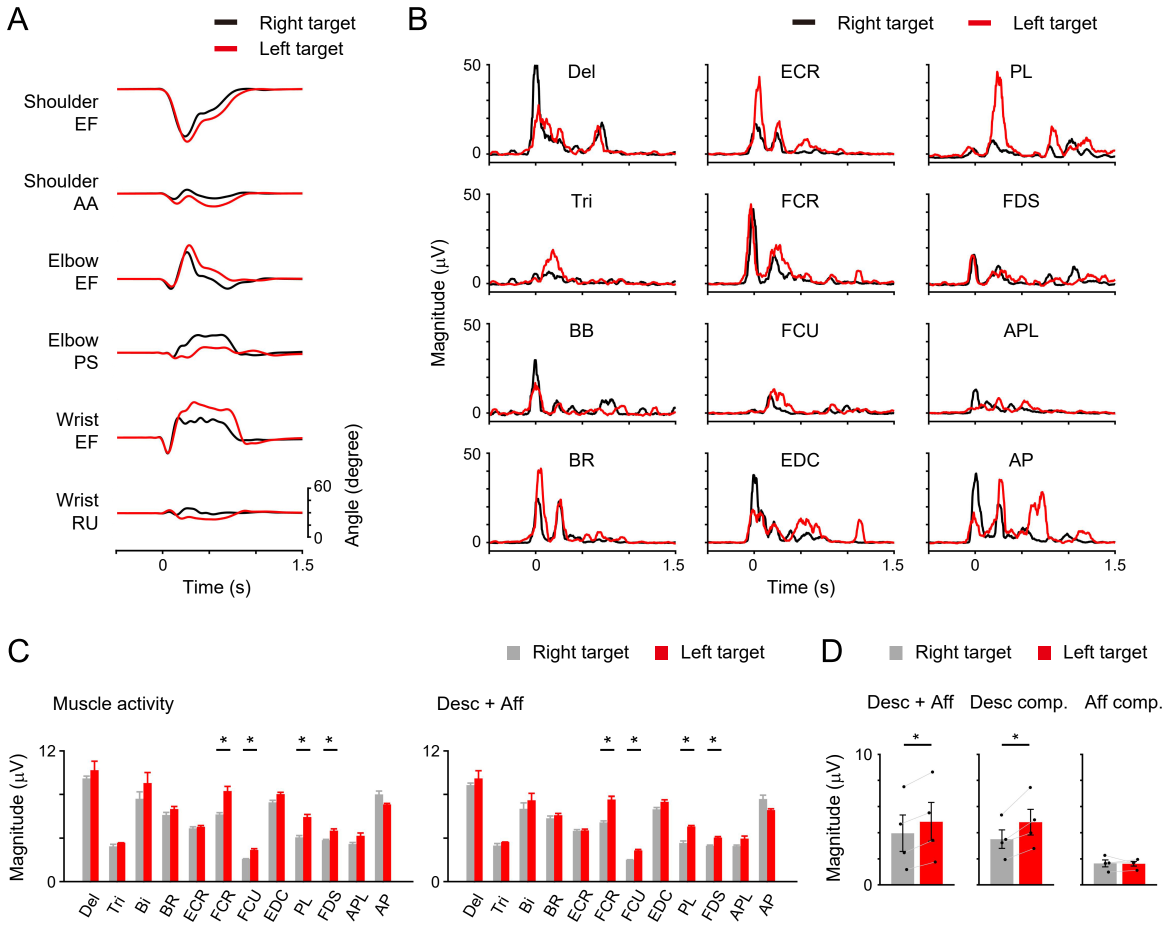

Fig. S6. The temporal pattern of joint kinematics and muscle activity differed across different movements. (*A*) Forelimb joint angles of the reaching and grasping movements to the right (black lines) and left targets (red lines). (*B*) Forelimb muscle activity of the reaching and grasping movements to the right (black lines) and left targets (red lines). (*C*) The size of the observed muscle activity and reconstruction using descending and afferent inputs for each muscle during reaching and grasping movements to the right (light gray) or left (red) targets (**P* < 0.05, unpaired two-tailed *t*-test). Data are the mean ± SEM. *P* values are described in Table S14. (*D*) The size of the reconstruction using descending and afferent inputs and each subcomponent for forearm flexors during reaching and grasping movements to the right (light gray) or left (red) targets (n = 4 sessions; **P* < 0.05, paired two-tailed *t*-test). The superimposed bar graphs show the mean ± SEM. *P* values are described in Table S15.

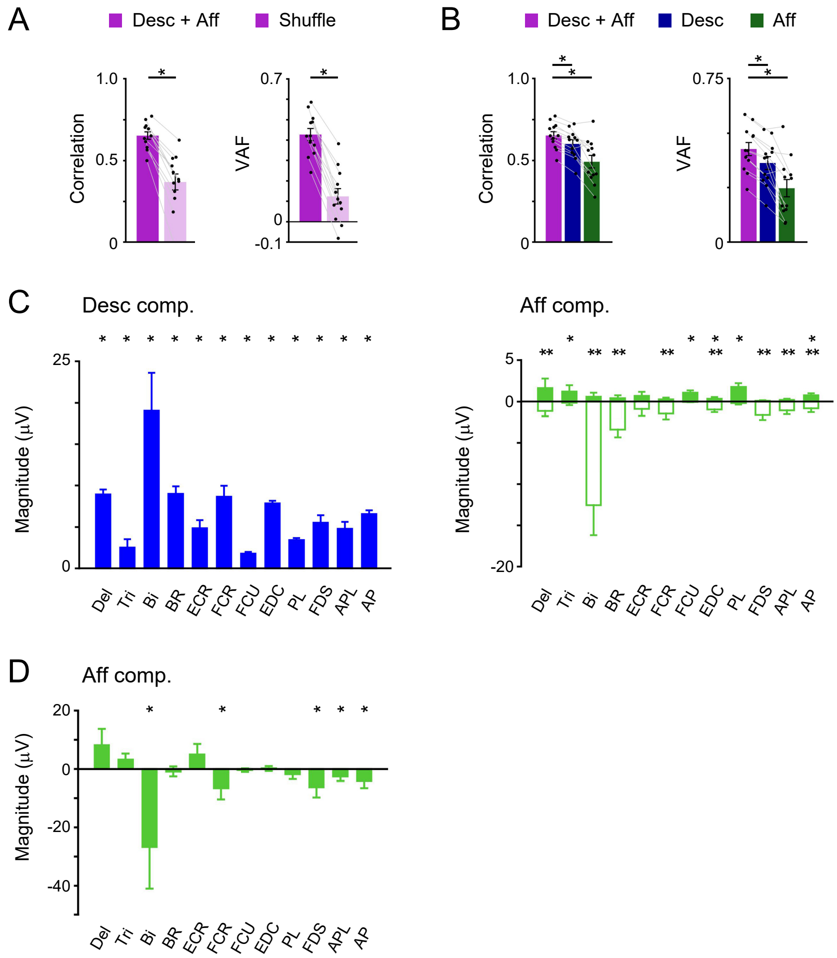

Fig. S7. The temporal dynamics of the sensorimotor convergence in the spinal motor neurons is a common feature across different movements. (*A*) Mean reconstruction accuracy. Correlation coefficients and VAFs between the observed and reconstructed traces (n = 12 muscles; $*P < 10^{-5}$, paired two-tailed *t*-test). The superimposed bar graphs show the mean ± SEM. *P* values are described in Table S16. (*B*) Correlation coefficients and VAFs between the observed and reconstructed traces (n = 12 muscles; $P < 10^{-4}$, one-way repeated-measures ANOVA, $*P < 0.05$, paired two-tailed *t*-test). The superimposed bar graphs show the mean ± SEM. *P* values are described in Table S17. (*C*) The size of descending and afferent components for each muscle. Asterisks indicate a significant difference from 0 (n = 4 sessions; $*P < 0.05$, unpaired two-tailed *t*-test for positive values; $**P < 0.05$, for negative values). Data are the mean ± SD. *P* values are described in Table S18. (*D*) The size of afferent components for each muscle in a period from the beginning of the reaching movement (55 to 100 ms around movement onset). Asterisks indicate a significant difference from 0 (n = 4 sessions; $*P < 0.05$, unpaired two-tailed *t*-test). Data are the mean ± SD. *P* values are described in Table S19.

Table S1. Statistics in Fig. 2C: Correlation coefficient and VAF between the observed and reconstructed traces

Paired two-tailed *t*-test

**Correlation coefficient**	n	P value
Desc + Aff vs. Shuffle (monkey T)	12	2.8×10^{-8}
Desc + Aff vs. Shuffle (monkey C)	10	3.3×10^{-6}

**VAF**	n	P value
Desc + Aff vs. Shuffle (monkey T)	12	5.8×10^{-9}
Desc + Aff vs. Shuffle (monkey C)	10	1.4×10^{-6}

Table S2. Statistics in Fig. 2F: Correlation coefficients and VAFs between the observed and reconstructed traces

One-way repeated-measures ANOVA

	F (DF)	P value
Correlation coefficient (monkey T)	$F(2, 11) = 20.26$	1.0×10^{-5}
Correlation coefficient (monkey C)	$F(2, 9) = 19.46$	0
VAF (monkey T)	$F(2, 11) = 23.41$	3.6×10^{-6}
VAF (monkey C)	$F(2, 9) = 35.21$	0

Paired two-tailed *t*-test

**Correlation coefficient (monkey T)**	n	P value
Desc + Aff vs. Desc	12	6.5×10^{-5}
Desc + Aff vs. Aff	12	7.6×10^{-5}

**Correlation coefficient (monkey C)**	n	P value
Desc + Aff vs. Desc	10	0.0059
Desc + Aff vs. Aff	10	4.4×10^{-4}

**VAF (monkey T)**	n	P value
Desc + Aff vs. Desc	12	1.7×10^{-4}
Desc + Aff vs. Aff	12	1.7×10^{-5}

**VAF (monkey C)**	n	P value
Desc + Aff vs. Desc	10	0.0013
Desc + Aff vs. Aff	10	6.7×10^{-5}

Table S3. Statistics in Fig. 3C: Onset times of the observed muscle activity, the reconstruction using descending and afferent inputs, and each subcomponent

One-way repeated-measures ANOVA

	F (DF)	P value
monkey T	$F(3, 11) = 56.35$	0
monkey C	$F(2, 7) = 28.23$	0

Paired two-tailed *t*-test

**Correlation coefficient (monkey T)**	n	P value
Observed vs. Desc + Aff	12	0.78
Observed vs. Desc comp.	12	0.74
Observed vs. Aff comp.	12	4.2×10^{-6}
Desc + Aff vs. Desc comp.	12	0.79
Desc + Aff vs. Aff comp.	12	7.1×10^{-6}
Desc comp.vs. Aff comp.	12	7.7×10^{-6}

**Correlation coefficient (monkey C)**	n	P value
Observed vs. Desc + Aff	10	4.4×10^{-4}
Observed vs. Desc comp.	10	0.087
Observed vs. Aff comp.	8	8.2×10^{-4}
Desc + Aff vs. Desc comp.	10	0.20
Desc + Aff vs. Aff comp.	8	6.3×10^{-4}
Desc comp.vs. Aff comp.	8	0.0016

Table S4. Statistics in Fig. 4B: Size of descending and afferent components

Unpaired two-tailed *t*-test

**Desc comp., positive (monkey T)**	n	P value
Del	17	4.5×10^{-16}
Tri	17	3.8×10^{-7}
Bi	17	1.0×10^{-8}
BR	17	3.0×10^{-10}
ECR	17	1.4×10^{-11}
FCR	17	3.2×10^{-13}
FCU	17	6.8×10^{-10}
EDC	17	2.0×10^{-12}
PL	17	6.9×10^{-10}
FDS	17	6.3×10^{-12}
APL	17	4.4×10^{-10}
AP	17	6.2×10^{-14}

**Desc comp., negative (monkey T)**	n	P value
Del	17	NaN
Tri	17	0.33
Bi	17	0.30
BR	17	0.33
ECR	17	NaN
FCR	17	0.33
FCU	17	NaN
EDC	17	NaN
PL	17	NaN
FDS	17	NaN
APL	17	NaN
AP	17	NaN

**Desc comp., positive (monkey C)**	n	P value
Del	7	1.6×10^{-4}

TriLo	7	6.4×10^{-7}
TriLa	7	8.3×10^{-5}
BR	7	2.8×10^{-5}
ECR	7	3.3×10^{-6}
FCU	7	1.6×10^{-5}
EDC	7	3.2×10^{-6}
FDP	7	4.6×10^{-5}
APL	7	1.7×10^{-4}
AP	7	2.3×10^{-4}

**Desc comp., negative (monkey C)**	n	P value
Del	7	NaN
TriLo	7	NaN
TriLa	7	0.26
BR	7	NaN
ECR	7	NaN
FCU	7	NaN
EDC	7	NaN
FDP	7	NaN
APL	7	NaN
AP	7	NaN

**Aff comp., positive (monkey T)**	n	P value
Del	17	4.1×10^{-7}
Tri	17	1.3×10^{-6}
Bi	17	0.016
BR	17	1.1×10^{-4}
ECR	17	3.5×10^{-6}
FCR	17	0.0079
FCU	17	6.9×10^{-9}
EDC	17	0.0048
PL	17	6.6×10^{-7}

FDS	17	0.018
APL	17	0.0062
AP	17	2.0×10^{-6}

**Aff comp., negative (monkey T)**	n	P value
Del	17	8.9×10^{-7}
Tri	17	0.0012
Bi	17	3.0×10^{-8}
BR	17	7.9×10^{-7}
ECR	17	1.6×10^{-6}
FCR	17	6.5×10^{-10}
FCU	17	2.2×10^{-7}
EDC	17	3.8×10^{-7}
PL	17	8.4×10^{-6}
FDS	17	6.3×10^{-9}
APL	17	3.6×10^{-6}
AP	17	6.6×10^{-5}

**Aff comp., positive (monkey C)**	n	P value
Del	7	0.024
TriLo	7	5.5×10^{-4}
TriLa	7	0.0011
BR	7	0.016
ECR	7	0.0052
FCU	7	0.0040
EDC	7	0.0014
FDP	7	0.0033
APL	7	0.0064
AP	7	0.013

**Aff comp., negative (monkey C)**	n	P value
Del	7	0.030
TriLo	7	0.048

TriLa	7	0.25
BR	7	0.048
ECR	7	0.039
FCU	7	0.010
EDC	7	0.12
FDP	7	0.26
APL	7	0.078
AP	7	0.10

NaN: Not a number

Table S5. Statistics in Fig. 5B: Size of subcomponents calculated from the activity in PMd, PMv, and M1

One-way repeated-measures ANOVA

	F (DF)	P value
monkey T	$F(2, 11) = 83.63$	0
monkey C	$F(2, 7) = 25.76$	0

Paired two-tailed *t*-test

**Normalized activity (monkey T)**	n	P value
PMd vs. PMv	12	8.6×10^{-7}
PMd vs. M1	12	1.4×10^{-6}
PMv vs. M1	12	2.6×10^{-6}

**Normalized activity (monkey C)**	n	P value
PMd vs. PMv	10	0.22
PMd vs. M1	10	5.8×10^{-4}
PMv vs. M1	10	7.2×10^{-5}

Table S6. Statistics in Fig. 6C: Size of afferent components in a period from the beginning of the reaching movement

Unpaired two-tailed *t*-test

**Aff comp. (monkey T)**	n	P value
ECR	17	3.8×10^{-4}
FCR	17	7.0×10^{-11}

**Aff comp. (monkey C)**	n	P value
TriLa	7	5.2×10^{-4}
BR	7	0.041

Table S7. Statistics in Fig. 7C: Onset times of the observed muscle activity, the reconstruction using descending and afferent inputs, and each subcomponent

One-way repeated-measures ANOVA

	F (DF)	P value
monkey T	F(3, 9) = 24.37	0

Paired two-tailed *t*-test

**Correlation coefficient (monkey T)**	n	P value
Observed vs. Desc + Aff	12	0.52
Observed vs. Desc comp.	12	0.29
Observed vs. Aff comp.	10	2.5×10^{-4}
Desc + Aff vs. Desc comp.	12	0.15
Desc + Aff vs. Aff comp.	10	7.9×10^{-5}
Desc comp.vs. Aff comp.	10	3.5×10^{-4}

Table S8. Statistics in Fig. S2C: Correlation coefficients and VAFs between the observed and reconstructed traces

One-way repeated-measures ANOVA

	F (DF)	P value
Correlation coefficient (monkey T)	$F(2, 11) = 26.9$	1.2×10^{-6}
Correlation coefficient (monkey C)	$F(2, 9) = 26.6$	4.2×10^{-6}
VAF (monkey T)	$F(2, 11) = 39.92$	4.8×10^{-8}
VAF (monkey C)	$F(2, 9) = 32.47$	1.1×10^{-6}

Paired two-tailed *t*-test

**Correlation coefficient (monkey T)**	n	P value
Desc + Aff vs. Desc + Shuffled Aff	12	2.8×10^{-8}
Desc + Aff vs. Aff + Shuffled Desc	12	5.4×10^{-5}

**Correlation coefficient (monkey C)**	n	P value
Desc + Aff vs. Desc + Shuffled Aff	10	2.3×10^{-4}
Desc + Aff vs. Aff + Shuffled Desc	10	8.7×10^{-5}

**VAF (monkey T)**	n	P value
Desc + Aff vs. Desc + Shuffled Aff	12	3.0×10^{-8}
Desc + Aff vs. Aff + Shuffled Desc	12	9.4×10^{-6}

**VAF (monkey C)**	n	P value
Desc + Aff vs. Desc + Shuffled Aff	10	1.8×10^{-4}
Desc + Aff vs. Aff + Shuffled Desc	10	2.5×10^{-5}

Table S9. Statistics in Fig. S2D: Proportion of descending inputs selected by the sparse linear regression model

One-way repeated-measures ANOVA

	F (DF)	P value
Proportion (monkey T)	F(2, 16) = 4204	0
Proportion (monkey C)	F(2, 6) = 1587	0

Paired two-tailed *t*-test

**Proportion (monkey T)**	n	P value
Desc + Aff vs. Desc + Shuffled Aff	17	7.8×10^{-5}
Desc + Aff vs. Aff + Shuffled Desc	17	2.7×10^{-20}
Desc + Shuffled Aff vs. Aff + Shuffled Desc	17	2.3×10^{-23}

**Proportion (monkey C)**	n	P value
Desc + Aff vs. Desc + Shuffled Aff	7	0.13
Desc + Aff vs. Aff + Shuffled Desc	7	1.6×10^{-8}
Desc + Shuffled Aff vs. Aff + Shuffled Desc	7	1.5×10^{-8}

Table S10. Statistics in Fig. S2E: Proportion of afferent inputs selected by the sparse linear regression model

One-way repeated-measures ANOVA

	F (DF)	P value
Proportion (monkey T)	F(2, 16) = 985	0
Proportion (monkey C)	F(2, 6) = 114	0

Paired two-tailed *t*-test

**Proportion (monkey T)**	n	P value
Desc + Aff vs. Aff + Shuffled Desc	17	0.0094
Desc + Aff vs. Desc + Shuffled Aff	17	2.0×10^{-16}
Desc + Shuffled Aff vs. Aff + Shuffled Desc	17	5.7×10^{-16}

**Proportion (monkey C)**	n	P value
Desc + Aff vs. Aff + Shuffled Desc	7	0.57
Desc + Aff vs. Desc + Shuffled Aff	7	5.3×10^{-5}
Desc + Shuffled Aff vs. Aff + Shuffled Desc	7	2.6×10^{-5}

Table S11. Statistics in Fig. S3B: VAFs between the observed and reconstructed traces for each 500-ms sliding window

Paired two-tailed *t*-test

**Desc + Aff vs. Desc (monkey T)**	n	P value
-0.5 s – 0 s	12	0.21
-0.45 s – 0.05 s	12	2.9×10^{-4}
-0.40 s – 0.1 s	12	2.1×10^{-5}
-0.35 s – 0.15 s	12	2.5×10^{-4}
-0.30 s – 0.2 s	12	1.0×10^{-5}
-0.25 s – 0.25 s	12	1.2×10^{-7}
-0.20 s – 0.3 s	12	3.1×10^{-7}
-0.15 s – 0.35 s	12	$4.2 \times 10\text{-}^{7}$
-0.10 s – 0.4 s	12	1.2×10^{-6}
-0.05 s – 0.45 s	12	1.6×10^{-7}
0 s – 0.5 s	12	1.3×10^{-6}
0.05 s – 0.55 s	12	2.2×10^{-4}
0.1 s – 0.6 s	12	0.0025
0.15 s – 0.65 s	12	0.0028
0.2 s – 0.7 s	12	0.0030
0.25 s – 0.75 s	12	2.4×10^{-4}
0.3 s – 0.8 s	12	1.4×10^{-5}
0.35 s – 0.85 s	12	9.0×10^{-7}
0.4 s – 0.9 s	12	3.3×10^{-7}
0.45 s – 0.95 s	12	7.4×10^{-8}
0.5 s – 1.0 s	12	1.8×10^{-8}

**Desc + Aff vs. Desc (monkey C)**	n	P value
-0.5 s – 0 s	10	0.24
-0.45 s – 0.05 s	10	0.042
-0.40 s – 0.1 s	10	0.0048
-0.35 s – 0.15 s	10	0.0010
-0.30 s – 0.2 s	10	7.2×10^{-4}

-0.25 s – 0.25 s	10	6.7×10^{-4}
-0.20 s – 0.3 s	10	3.8×10^{-4}
-0.15 s – 0.35 s	10	3.9×10^{-4}
-0.10 s – 0.4 s	10	5.4×10^{-4}
-0.05 s – 0.45 s	10	0.0014
0 s – 0.5 s	10	4.4×10^{-4}
0.05 s – 0.55 s	10	3.4×10^{-4}
0.1 s – 0.6 s	10	3.7×10^{-4}
0.15 s – 0.65 s	10	4.7×10^{-5}
0.2 s – 0.7 s	10	1.0×10^{-5}
0.25 s – 0.75 s	10	8.3×10^{-5}
0.3 s – 0.8 s	10	8.0×10^{-4}
0.35 s – 0.85 s	10	9.4×10^{-4}
0.4 s – 0.9 s	10	0.0014
0.45 s – 0.95 s	10	5.6×10^{-4}
0.5 s – 1.0 s	10	2.6×10^{-5}

Paired two-tailed *t*-test

**Desc + Aff vs. Aff (monkey T)**	n	P value
-0.5 s – 0 s	12	7.1×10^{-9}
-0.45 s – 0.05 s	12	3.3×10^{-6}
-0.40 s – 0.1 s	12	1.9×10^{-6}
-0.35 s – 0.15 s	12	2.8×10^{-6}
-0.30 s – 0.2 s	12	4.3×10^{-5}
-0.25 s – 0.25 s	12	4.5×10^{-5}
-0.20 s – 0.3 s	12	4.2×10^{-5}
-0.15 s – 0.35 s	12	2.7×10^{-5}
-0.10 s – 0.4 s	12	8.2×10^{-6}
-0.05 s – 0.45 s	12	1.6×10^{-5}
0 s – 0.5 s	12	4.2×10^{-8}
0.05 s – 0.55 s	12	1.8×10^{-4}
0.1 s – 0.6 s	12	0.0028

0.15 s – 0.65 s	12	0.015
0.2 s – 0.7 s	12	0.037
0.25 s – 0.75 s	12	0.036
0.3 s – 0.8 s	12	0.012
0.35 s – 0.85 s	12	0.0067
0.4 s – 0.9 s	12	0.0048
0.45 s – 0.95 s	12	2.6×10^{-4}
0.5 s – 1.0 s	12	0.0043

**Desc + Aff vs. Aff (monkey C)**	n	P value
-0.5 s – 0 s	10	3.7×10^{-7}
-0.45 s – 0.05 s	10	1.1×10^{-4}
-0.40 s – 0.1 s	10	2.3×10^{-4}
-0.35 s – 0.15 s	10	7.6×10^{-5}
-0.30 s – 0.2 s	10	1.6×10^{-4}
-0.25 s – 0.25 s	10	4.1×10^{-4}
-0.20 s – 0.3 s	10	3.3×10^{-4}
-0.15 s – 0.35 s	10	2.4×10^{-4}
-0.10 s – 0.4 s	10	2.8×10^{-4}
-0.05 s – 0.45 s	10	2.1×10^{-5}
0 s – 0.5 s	10	1.6×10^{-7}
0.05 s – 0.55 s	10	9.5×10^{-6}
0.1 s – 0.6 s	10	1.4×10^{-5}
0.15 s – 0.65 s	10	7.0×10^{-6}
0.2 s – 0.7 s	10	8.6×10^{-6}
0.25 s – 0.75 s	10	3.6×10^{-6}
0.3 s – 0.8 s	10	2.9×10^{-5}
0.35 s – 0.85 s	10	1.3×10^{-4}
0.4 s – 0.9 s	10	1.1×10^{-4}
0.45 s – 0.95 s	10	7.5×10^{-5}
0.5 s – 1.0 s	10	1.1×10^{-4}

Table S12. Statistics in Fig. S4: Proportion of PMd, PMv, and M1 inputs selected by the sparse linear regression model

One-way repeated-measures ANOVA

	F (DF)	P value
monkey T	$F(2, 16) = 55.05$	0
monkey C	$F(2, 6) = 9.32$	0.0036

Paired two-tailed *t*-test

**Proportion (monkey T)**	n	P value
PMd vs. PMv	17	0.013
PMd vs. M1	17	3.5×10^{-7}
PMv vs. M1	17	2.0×10^{-8}

**Proportion (monkey C)**	n	P value
PMd vs. PMv	7	0.52
PMd vs. M1	7	0.0011
PMv vs. M1	7	0.029

Table S13. Statistics in Fig. S5: Size of afferent components in a period from the beginning of the reaching movement

Unpaired two-tailed *t*-test

**Aff comp. (monkey T)**	n	P value
Del	17	1.4×10^{-7}
Tri	17	5.4×10^{-7}
Bi	17	1.3×10^{-7}
BR	17	0.038
ECR	17	3.8×10^{-4}
FCR	17	7.0×10^{-11}
FCU	17	0.21
EDC	17	0.055
PL	17	0.0021
FDS	17	1.4×10^{-9}
APL	17	0.015
AP	17	0.036

**Aff comp. (monkey C)**	n	P value
Del	7	0.28
TriLo	7	0.47
TriLa	7	5.2×10^{-4}
BR	7	0.041
ECR	7	0.88
FCU	7	0.13
EDC	7	1.3×10^{-4}
FDP	7	0.011
APL	7	0.33
AP	7	0.016

Table S14. Statistics in Fig. S6C: Size of the observed and reconstructed muscle activity

Unpaired two-tailed *t*-test

**Muscle activity**	n	P value
Del	17, 4	0.30
Tri	17, 4	0.55
Bi	17, 4	0.37
BR	17, 4	0.43
ECR	17, 4	0.76
FCR	17, 4	8.5×10^{-4}
FCU	17, 4	4.4×10^{-4}
EDC	17, 4	0.21
PL	17, 4	0.0012
FDS	17, 4	0.018
APL	17, 4	0.11
AP	17, 4	0.29

**Desc + Aff**	n	P value
Del	17, 4	0.37
Tri	17, 4	0.55
Bi	17, 4	0.56
BR	17, 4	0.65
ECR	17, 4	0.89
FCR	17, 4	4.1×10^{-4}
FCU	17, 4	2.7×10^{-4}
EDC	17, 4	0.20
PL	17, 4	0.0090
FDS	17, 4	0.011
APL	17, 4	0.10
AP	17, 4	0.24

Table S15. Statistics in Fig. S6D: Size of the reconstructed muscle activity and descending and afferent components

Paired two-tailed *t*-test

	n	P value
Desc + Aff	4	0.026
Desc comp.	4	0.0046
Aff comp.	4	0.90

Table S16. Statistics in Fig. S7A: Correlation coefficient and VAF between the observed and reconstructed traces

Paired two-tailed *t*-test

**Correlation coefficient**	n	P value
Desc + Aff vs. Shuffle (monkey T)	12	2.4×10^{-6}

**VAF**	n	P value
Desc + Aff vs. Shuffle (monkey T)	12	3.8×10^{-8}

Table S17. Statistics in Fig. S7B: Correlation coefficients and VAFs between the observed and reconstructed traces

One-way repeated-measures ANOVA

	F (DF)	P value
Correlation coefficient (monkey T)	$F(2, 11) = 40.62$	4.1×10^{-8}
VAF (monkey T)	$F(2, 11) = 40.98$	3.8×10^{-8}

Paired two-tailed *t*-test

**Correlation coefficient (monkey T)**	n	P value
Desc + Aff vs. Desc	12	4.9×10^{-7}
Desc + Aff vs. Aff	12	1.2×10^{-5}

**VAF (monkey T)**	n	P value
Desc + Aff vs. Desc	12	2.6×10^{-7}
Desc + Aff vs. Aff	12	6.9×10^{-6}

Table S18. Statistics in Fig. S7C: Size of descending and afferent components

Unpaired two-tailed *t*-test

**Desc comp., positive (monkey T)**	n	P value
Del	4	8.9×10^{-5}
Tri	4	0.016
Bi	4	0.0037
BR	4	3.0×10^{-4}
ECR	4	0.0023
FCR	4	9.9×10^{-4}
FCU	4	6.0×10^{-4}
EDC	4	1.7×10^{-5}
PL	4	1.1×10^{-4}
FDS	4	0.0011
APL	4	0.0015
AP	4	8.5×10^{-5}

**Desc comp., negative (monkey T)**	n	P value
Del	4	NaN
Tri	4	NaN
Bi	4	NaN
BR	4	NaN
ECR	4	NaN
FCR	4	NaN
FCU	4	NaN
EDC	4	NaN
PL	4	NaN
FDS	4	NaN
APL	4	NaN
AP	4	NaN

**Aff comp., positive (monkey T)**	n	P value
Del	4	0.069

Tri	4	0.047
Bi	4	0.098
BR	4	0.092
ECR	4	0.066
FCR	4	0.11
FCU	4	0.0042
EDC	4	0.040
PL	4	0.0044
FDS	4	0.057
APL	4	0.088
AP	4	0.011

**Aff comp., negative (monkey T)**	n	P value
Del	4	0.033
Tri	4	0.34
Bi	4	0.0063
BR	4	0.0052
ECR	4	0.12
FCR	4	0.030
FCU	4	0.17
EDC	4	0.0093
PL	4	0.11
FDS	4	0.013
APL	4	0.019
AP	4	0.044

Table S19. Statistics in Fig.S7D: Size of afferent components in a period from the beginning of the reaching movement

Unpaired two-tailed *t*-test

**Aff comp. (monkey T)**	n	P value
Del	4	0.064
Tri	4	0.052
Bi	4	0.033
BR	4	0.40
ECR	4	0.068
FCR	4	0.041
FCU	4	0.34
EDC	4	0.68
PL	4	0.12
FDS	4	0.039
APL	4	0.050
AP	4	0.039
